# Supplementary material for: Defining the biogeographical map and potential bacterial translocation of microbiome in human ‘surface organs’
Source: Nat Commun. 2024 Jan 10;15:427. doi: 10.1038/s41467-024-44720-6 (PMC10781665; doi:10.1038/s41467-024-44720-6)
Supplement: Supplementary file 1 — Supplementary Information [file 41467_2024_44720_MOESM1_ESM.pdf]

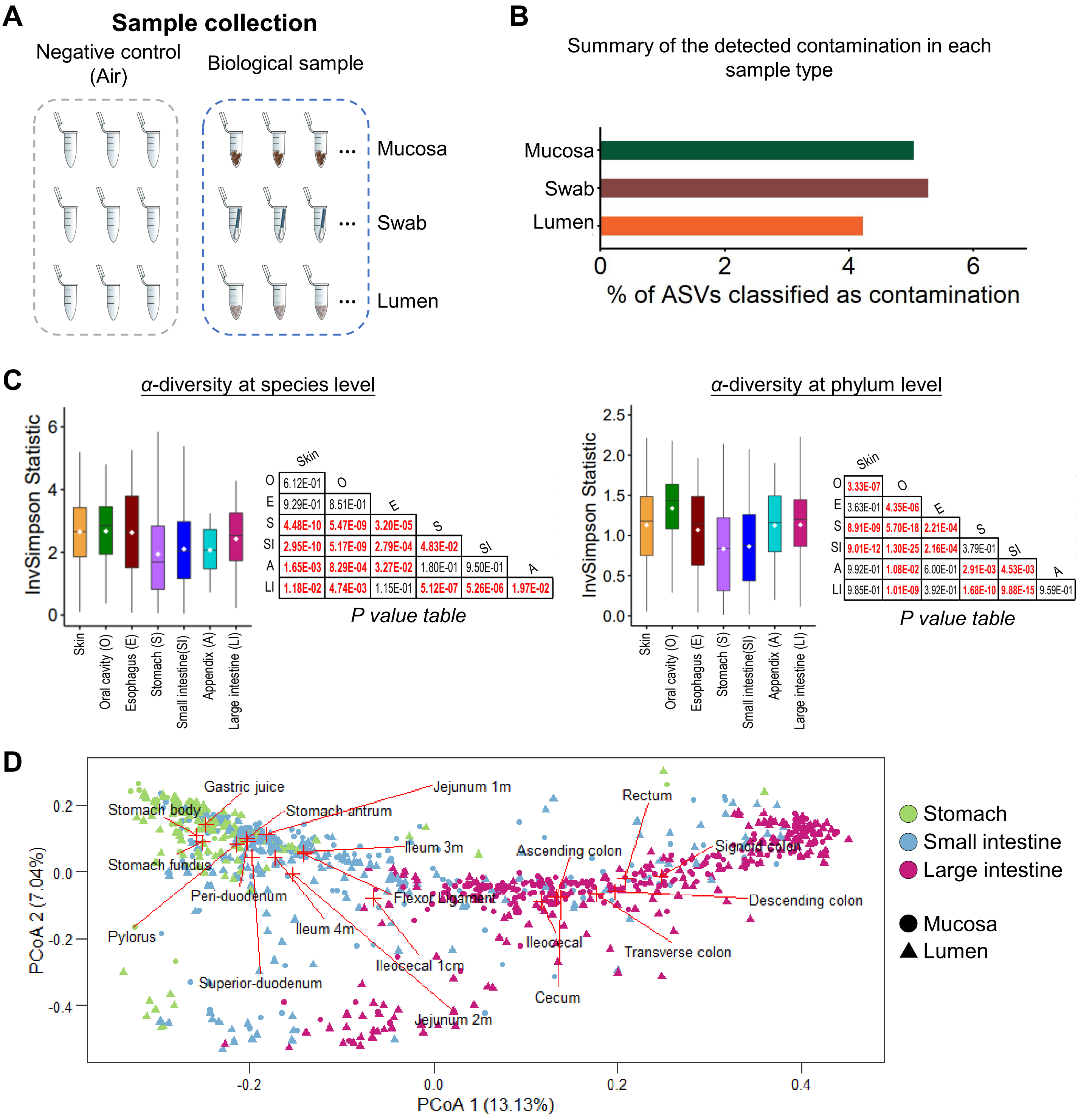

P value table (α-diversity at species level)

	Skin	O	E	S	SI	A
O	6.12E-01					
E	9.29E-01	8.51E-01				
S	4.48E-10	5.47E-09	3.20E-05			
SI	2.95E-10	5.17E-09	2.79E-04	4.83E-02		
A	1.65E-03	8.29E-04	3.27E-02	1.80E-01	9.50E-01	
LI	1.18E-02	4.74E-03	1.15E-01	5.12E-07	5.26E-06	1.97E-02

P value table (α-diversity at phylum level)

	Skin	O	E	S	SI	A
O	3.33E-07					
E	3.63E-01	4.35E-06				
S	8.91E-09	5.70E-18	2.21E-04			
SI	9.01E-12	1.30E-25	2.16E-04	3.79E-01		
A	9.92E-01	1.08E-02	6.00E-01	2.91E-03	4.53E-03	
LI	9.85E-01	1.01E-09	3.92E-01	1.68E-10	9.88E-15	9.59E-01

Figure S1. (A) Overview of sample collection including environmental negative controls (*n*=9) and real biological samples. The negative controls were divided into three groups (*n*=3 per group). The subsequent laboratory treatment (DNA extraction, library preparation, etc.) was the same as the biological samples of different sample types (surface sample: swab and mucosa; lumen). (**B**) Percentage of ASVs classified as contamination in each sample type. (**C**) *α*-diversity of samples was grouped by organs (*n*=328, 198, 110, 150, 363, 32, 427 for skin, oral cavity, esophagus, stomach, small intestine, appendix, and large intestine, respectively) and measured using the relative inverse Simpson index. Boxplots were colored by surface organs. *P* values were determined using two-sided Wilcoxon signed-rank test. Data are shown as Box and whisker plots to represent the median (center line), quartiles (box), and range (whiskers) of the *α*-diversity for each community, excluding outliers (points outside 1.5 times the interquartile range). (**D**) Beta diversity analysis for each section of stomach, small intestine, and large intestine. The symbol "+" represents the central location of samples from the same region.

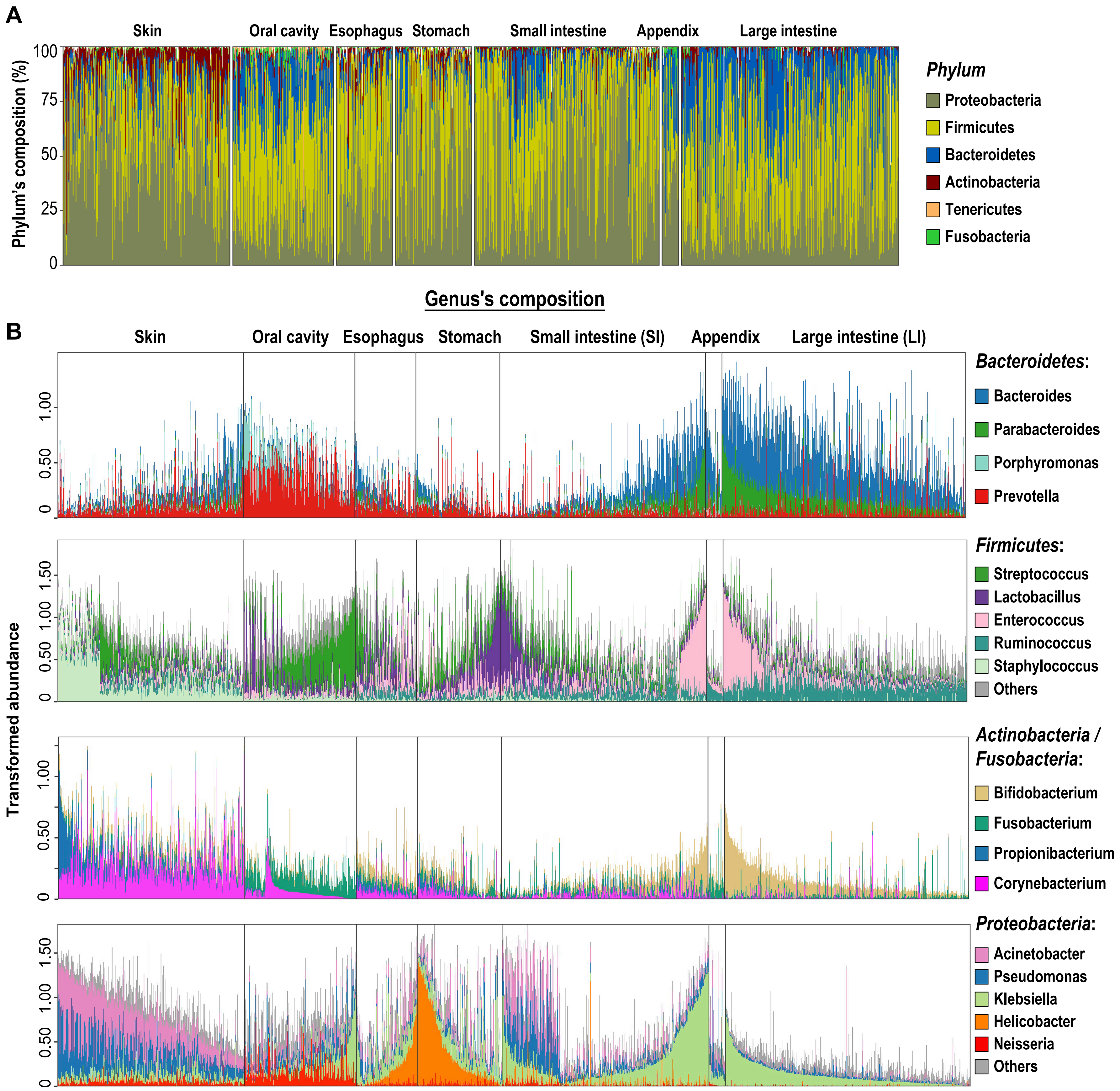

Figure S2. (**A**) Abundances of major phyla in seven organs. The x-axis represented samples grouped by organs. (**B**) Abundance distributions of the top abundant genera, with relative abundance >0.5% and prevalence >75%, and were organized by their belonged phylum. The x-axis represented samples grouped by organs, and hierarchical clustering was performed within each organ. The y-axis indicated transformed abundance (arcsine square root).

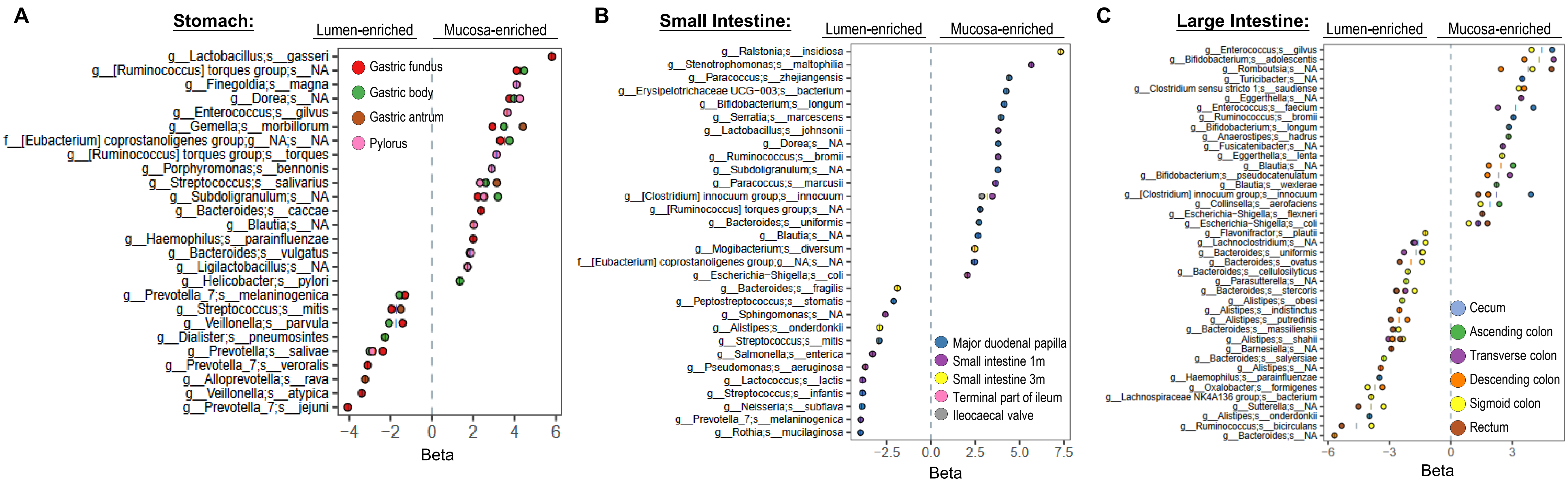

Figure S3. Significant mucosa-enriched and lumen/juice-enriched microbes in different regions of (**A**) stomach, (**B**) small or (**C**) large intestines, measured using logistic regression model. 16S full-length data were used to obtain the lumen/mucosa-enriched species. Beta values represented the magnitude of difference in relative abundance between paired luminal and mucosal samples, and the degree of consistency among subjects. Points were coloured by regions. FDR<0.05

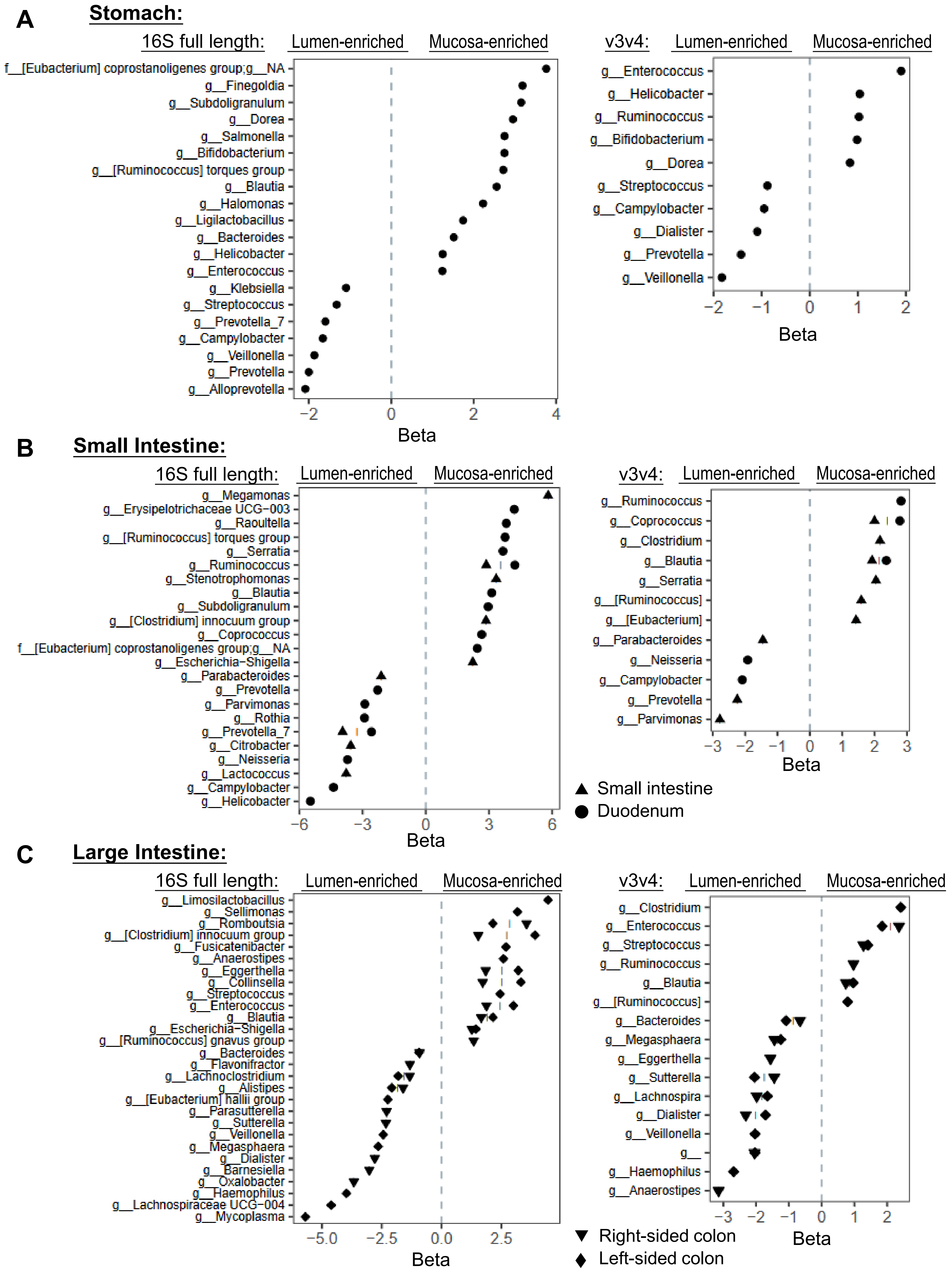

Figure S4. Consistency of lumen/mucosa-enriched bacteria between 16S full-length dataset (*left*) and v3v4 dataset (*right*) in (**A**) Stomach, (**B**) Small intestine, and (**C**) large intestine

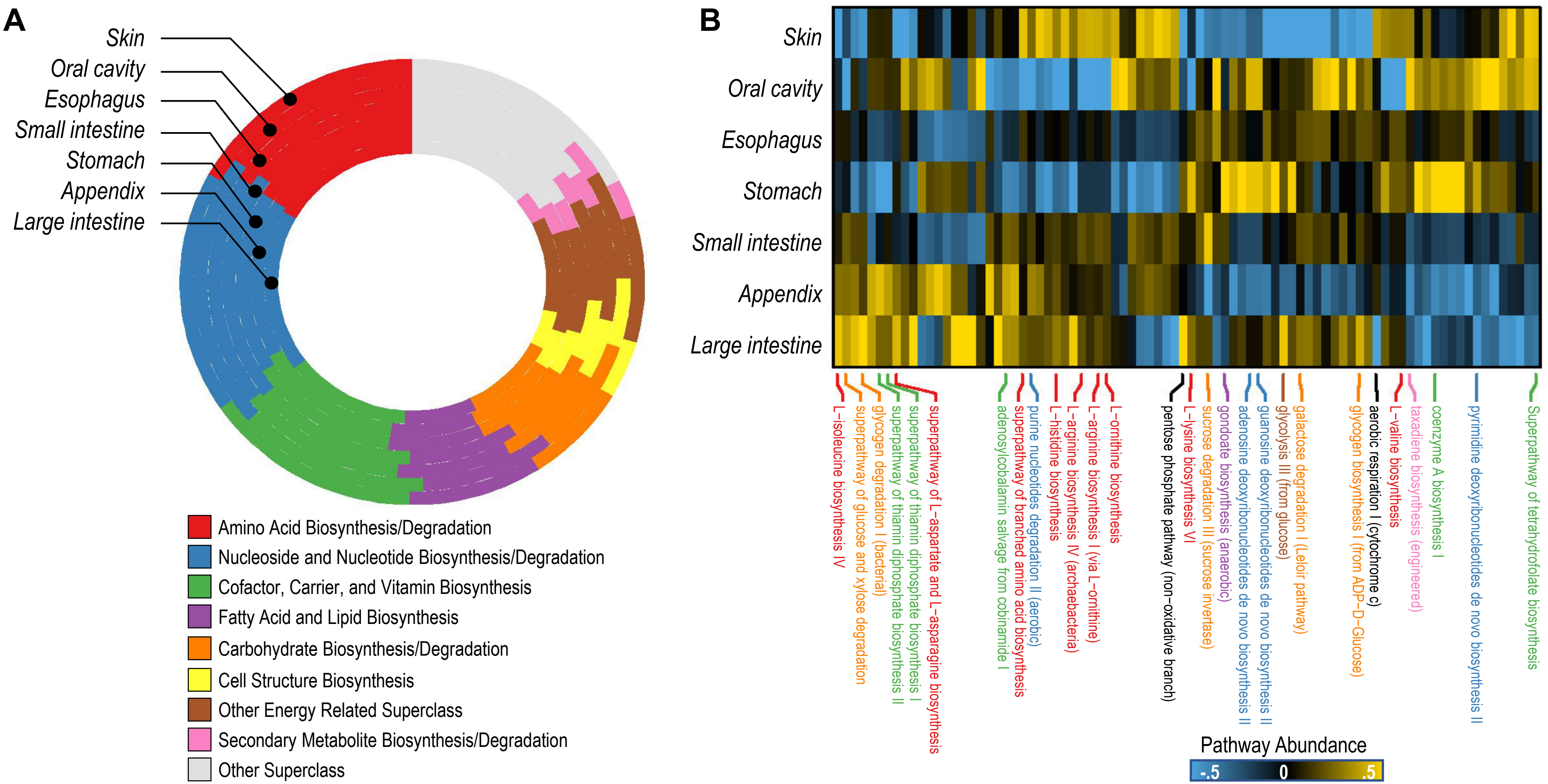

Figure S5. Functional characteristics of microbiome among seven surface organs. (**A**) Relative abundance of predicted functional categories in each organ. The plot was coloured by functional categories (super-class of pathway). (**B**) Comparative analysis among organs and hierarchical clustering analysis based on the abundance of predicted pathways. Only significantly altered pathways (FDR<0.05) were included and selected pathways were coloured by functional categories. *P* values were adjusted using FDR correction.

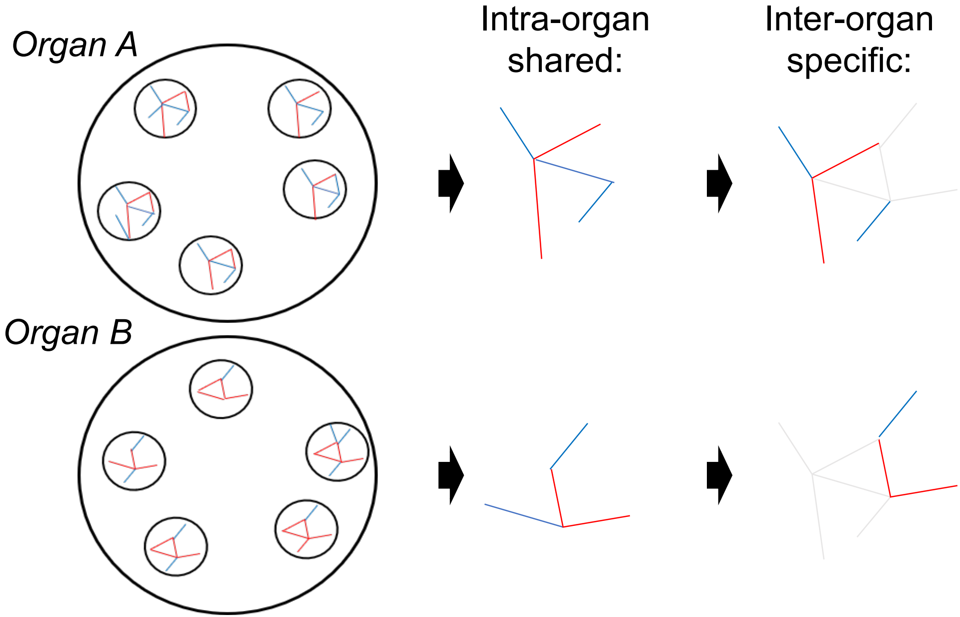

Figure S6. Shown was schematic of the selection of organ-specific correlation. Microbial correlations per region (*n*=53) were calculated using SparCC or SECOM method. To obtain organ-specific correlation, we compared microbial correlation among multiple intra-organ regions. Only correlation with the same direction (positive/negative correlation, *P*<0.05) in all intra-organ regions were selected for further evaluation. Organ-specific correlation was included if it fulfilled any of the following two conditions: 1) the difference in correlations between organs >0.6; 2) the correlation with strength >0.6 was only present in a specific organ.

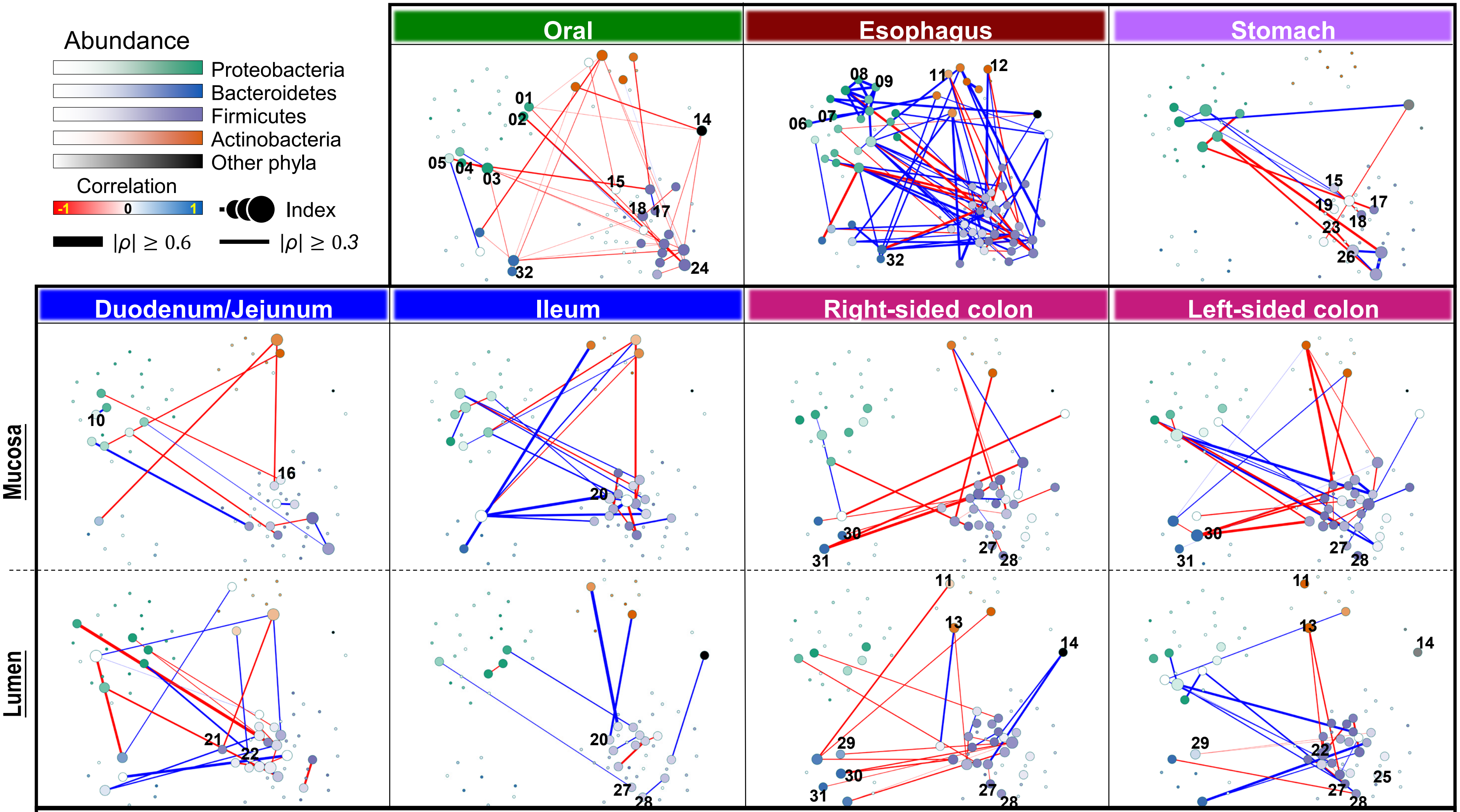

Nodes represent taxa at genus level

01	*Actinobacillus*	06	*Neisseria*	11	*Bifidobacterium*	16	*[Ruminococcus]*	21	*Lactobacillus*	26	*Clostridium*	31	*Parabacteroides*
02	*Aggregatibacter*	07	*Stenotrophomonas*	12	*Corynebacterium*	17	*Veillonella*	22	*Dorea*	27	*Faecalibacterium*	32	*Porphyromonas*
03	*Campylobacter*	08	*Ralstonia*	13	*Collinsella*	18	*Streptococcus*	23	*Dialister*	28	*Eubacterium*		
04	*Haemophilus*	09	*Agrobacterium*	14	*Fusobacterium*	19	*Blautia*	24	*Peptostreptococcus*	29	*Prevotella*		
05	*Klebsiella*	10	*Sutterella*	15	*Enterococcus*	20	*Ruminococcus*	25	*Parvimonas*	30	*Bacteroides*		

Figure S7. The organ-specific microbial network in the digestive organs. Mucosa and lumen of the lower digestive tract were analyzed separately. Correlation coefficients were estimated and corrected for compositional effects using SECON algorithm. Representative genera were denoted by node numbers accordingly. Nodes classified as members of the same phylum were grouped and coloured by their belonged phylum. Node size and colour gradient represented node index (normalized node degree) and average taxa abundance, respectively.

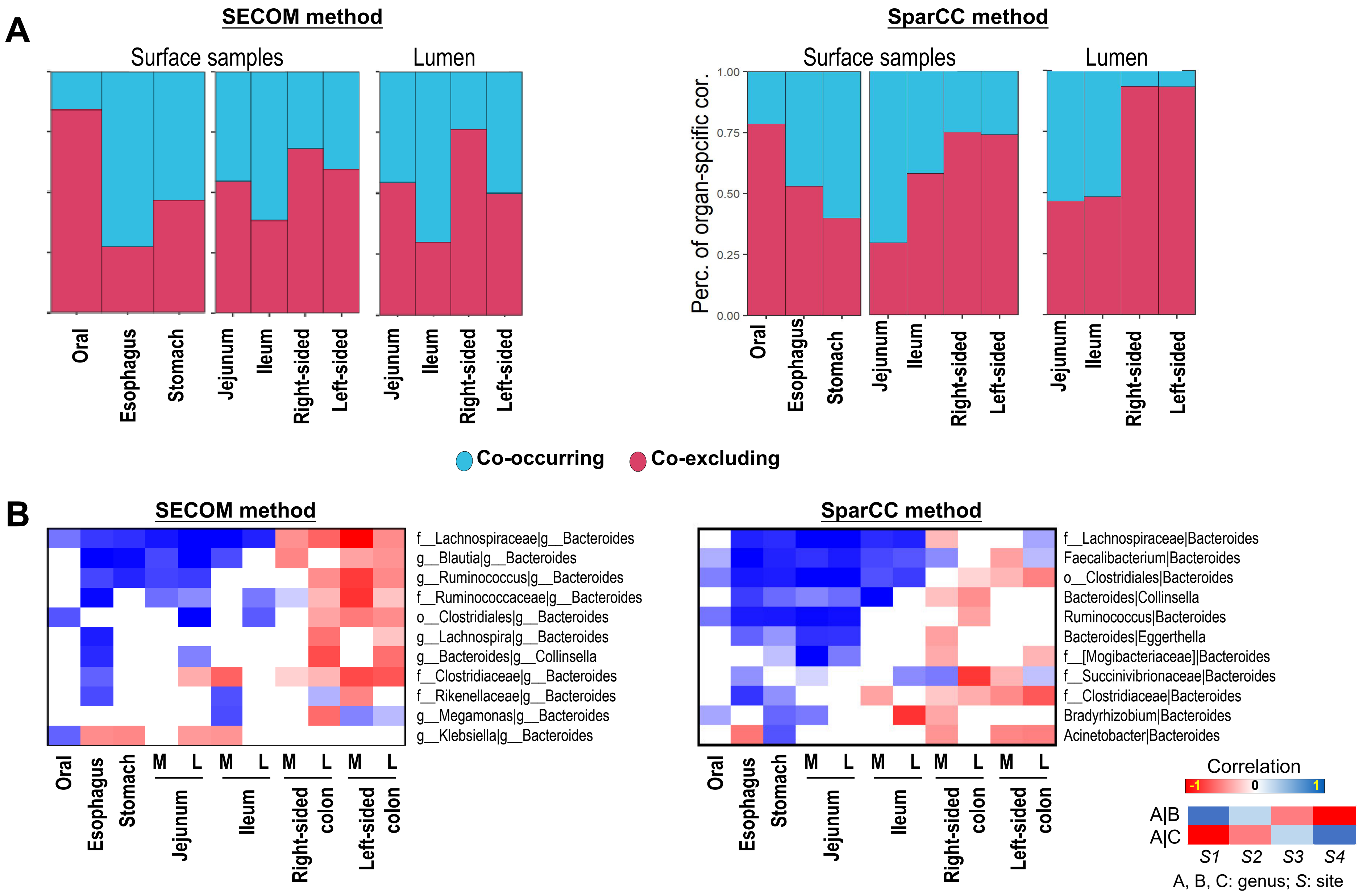

Figure S8. (**A**) Overall microbial correlation (co-occurrent and co-exclusive) in surface and lumen samples of each organ was displayed. The microbial correlations were computed by SECOM (*left*) or SparCC (*right*). (**B**) Co-occurrent and co-exclusive relationships between *Bacteroides* and other microbes identified by by SECOM (*left*) or SparCC (*right*) in different surface organs were displayed.

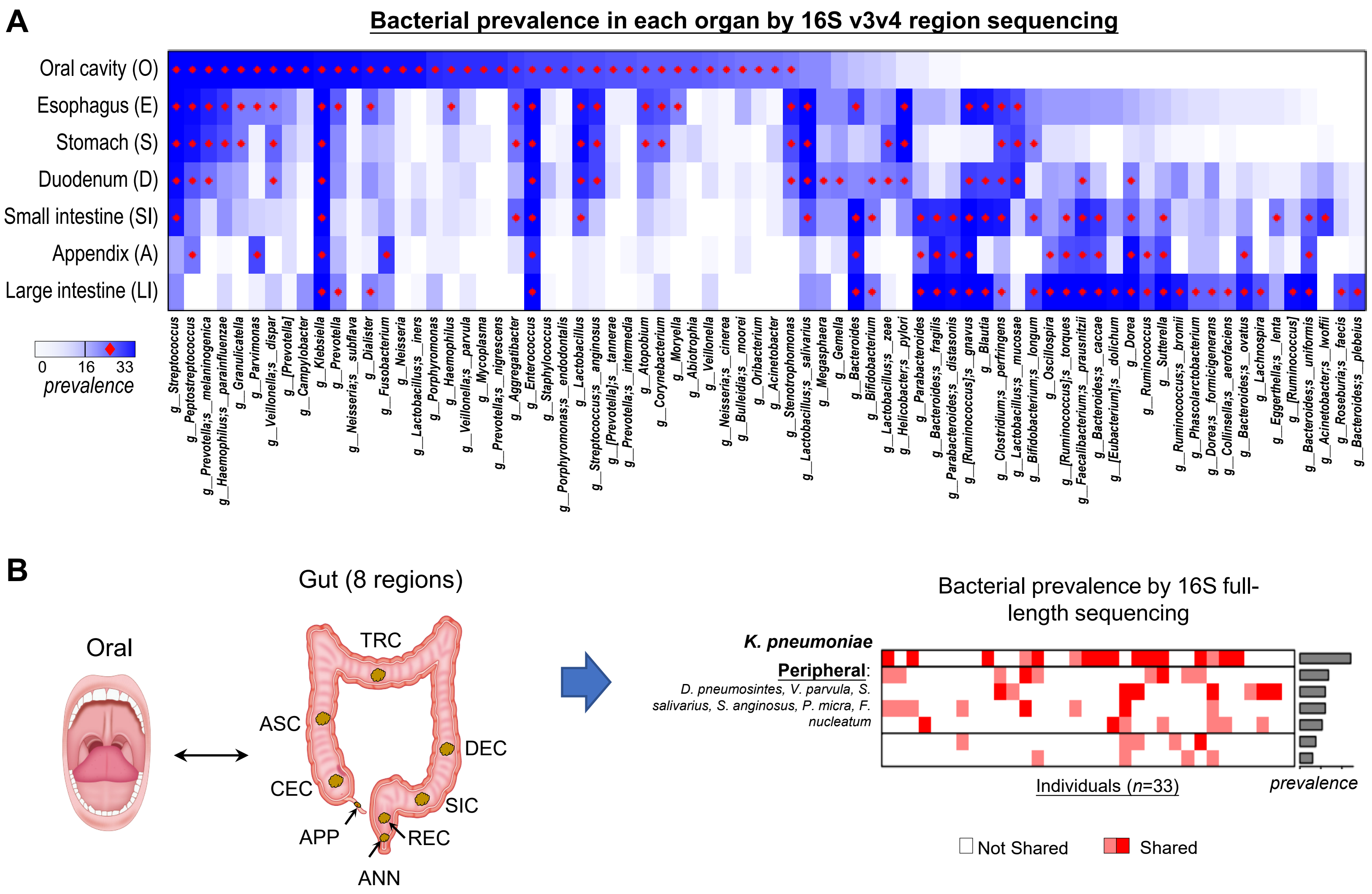

Figure S9. (**A**) Bacterial prevalence in each organ by 16S v3v4 region sequencing. ASVs with relative abundance >0.1% were considered as present on the organ. (**B**) 16S full-length ASVs that simultaneously present in oral and gut from the same individual. Areas labelled in red represent the presence of ASV on all the organs from the same individual (relative abundance >0.1% for all). The light red color represents there are only one type of ASV of a particular species shared among organs from the same individuals, while the dark red color represents there are >1 types of ASVs (of that same species) shared among organs from the same individuals.

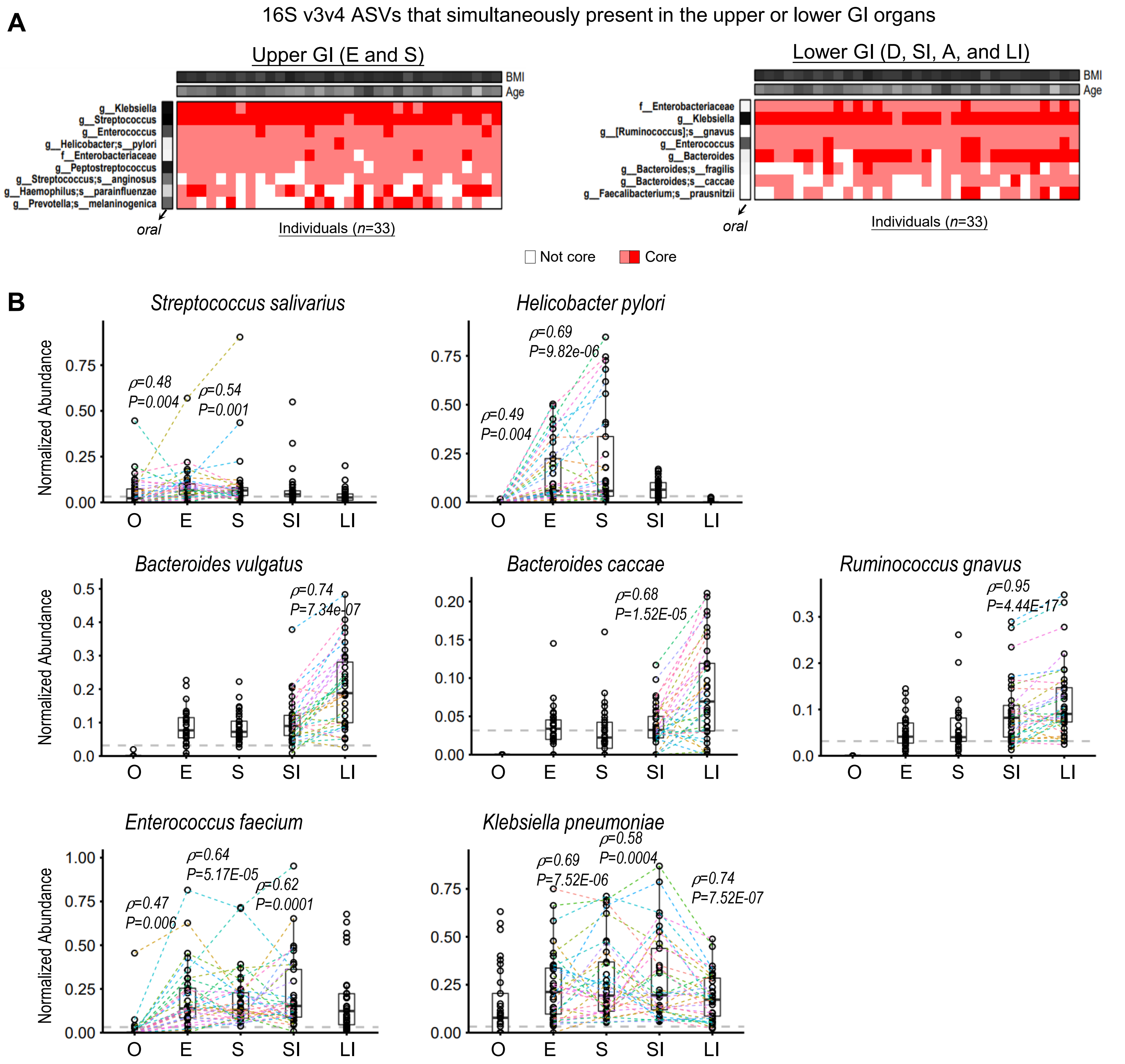

Figure S10. (**A**) ASVs simultaneously present in all the organs of intra-individual upper GI (left panel) or intra-individual lower GI tract (right panel). Areas labelled in red represent the presence of ASV on all the organs from the same individual (relative abundance >0.1% for all). The light red color represents there are only one type of ASV of a particular species shared among organs from the same individuals, while the dark red color represents there are >1 types of ASVs (of that same species) shared among organs from the same individuals. The bacterial prevalence in the oral cavity was displayed on left side of the plot. (**B**) The abundance changes of selected bacteria, with dash line links the same individual (*P*<0.05, correlation analysis). Data are shown as Box and whisker plots (A2) to represent the median (center line), quartiles (box), range (whiskers), and outliers (points outside 1.5 times the interquartile range). Two-tailed Spearman correlation, Partial spearman correlation, and two-tailed Pearson correlation were used simultaneously.

Table S1. Characteristics of 33 human subjects

No.	Age	Gender	BMI	Ethnicity	Dietary (staple food)	Resident place	Sampling duration after death (Min)	Hospitalization (Day)	Hospitalization group	Antibiotic uses (Yes: 1; No: 0)	Types of antibiotic use	ICU (Yes: 1; No: 0)	High Blood Pressure	Underlying disease	Cause of death
S001	43	Male	24.50	Han Chinese	noodles,rice	Shaanxi province	90	>30 days	>1 week	1	β-lactams	1	N	N	cerebral hemorrhage caused by a fall at the construction site due to mis-operation.
S002	51	Male	23.00	Han Chinese	noodles	Shaanxi province	95	2 days	<1 week	0	N	0	Y	N	cerebral hemorrhage caused by a fall down the stairs of a high-rise residential building
S003	49	Male	24.20	Han Chinese	noodles	Shaanxi province	85	1 day	no	0	N	0	N	N	cerebral trauma caused by car accident
S004	53	Male	20.90	Han Chinese	noodles	Shaanxi province	80	2 days	<1 week	0	N	0	N	N	cerebral hemorrhage caused by traumatic brain injury
S005	67	Male	22.10	Han Chinese	noodles	Shaanxi province	90	>10 days	>1 week	1	β-lactams	1	Y	N	cerebral trauma caused by car accident
S006	63	Male	23.70	Han Chinese	noodles	Shaanxi province	95	2 days	<1 week	1	β-lactams	0	Y	N	brain injury caused by a fall while walking
S007	46	Female	21.10	Han Chinese	noodles	Shaanxi province	80	3 days	<1 week	1	β-lactams	0	Y	N	cerebral trauma caused by car accident
S008	56	Male	23.10	Han Chinese	noodles	Shaanxi province	75	>30 days	>1 week	1	β-lactams	1	N	N	cerebral hemorrhage caused by car accident
S009	59	Male	20.30	Han Chinese	noodles	Shaanxi province	85	8 days	>1 week	1	β-lactams	1	Y	N	brain trauma caused by a fall on the step
S010	48	Male	24.30	Han Chinese	noodles	Shaanxi province	75	1 day	no	0	N	0	Unknown	N	cerebral hemorrhage caused by car accident
S011	47	Male	21.70	Han Chinese	noodles	Shaanxi province	75	1 day	no	0	N	0	Y	N	brainstem hemorrhage from a fall on a high step
S012	40	Male	26.20	Han Chinese	noodles	Shaanxi province	85	1 day	no	0	N	0	Y	N	brainstem hemorrhage caused by car accident
S013	37	Male	24.10	Han Chinese	noodles,rice	Shaanxi province	75	7 days	<1 week	1	β-lactams	0	Y	N	cerebral hemorrhage caused by fall from a height
S014	48	Male	22.50	Han Chinese	noodles	Shaanxi province	90	5 days	<1 week	1	β-lactams	0	N	N	cerebral hemorrhage caused by car accident
S015	34	Male	22.50	Han Chinese	noodles,rice	Shaanxi province	95	>90 days	>1 week	1	β-lactams	1	Unknown	N	traumatic brain injury by accident
S016	49	Male	25.30	Han Chinese	noodles	Shaanxi province	80	6 days	<1 week	1	β-lactams	0	Y	N	traumatic brain injury by fall on the stairs of a high-rise building
S017	42	Male	22.60	Han Chinese	noodles	Shaanxi province	85	1 day	no	0	N	0	N	N	cerebral hemorrhage caused by traumatic brain injury
S018	39	Male	24.60	Han Chinese	noodles	Shaanxi province	75	1 day	no	0	N	0	N	N	traumatic brain injury by accident
S019	61	Male	23.30	Han Chinese	noodles	Shaanxi province	80	1 day	no	0	N	0	Y	N	cerebral hemorrhage caused by high-altitude falling
S020	74	Male	24.90	Han Chinese	noodles	Shaanxi province	85	1 day	no	0	N	0	Y	N	cerebral hemorrhage after an accidental fall
S021	54	Male	23.70	Han Chinese	noodles,rice	Shaanxi province	75	3 days	<1 week	1	β-lactams	0	N	N	cerebral hemorrhage caused by car accident
S022	47	Male	24.50	Han Chinese	noodles	Shaanxi province	85	2 days	<1 week	1	β-lactams	0	Y	N	cerebral hemorrhage caused by car accident
S023	66	Male	24.90	Han Chinese	noodles	Shaanxi province	85	1 day	no	0	N	0	N	N	brainstem hemorrhage due to accidental injury
S024	45	Male	19.70	Han Chinese	noodles	Shaanxi province	90	3 days	<1 week	1	β-lactams	0	Y	N	cerebral hemorrhage caused by car accident
S025	51	Male	24.20	Han Chinese	noodles	Shaanxi province	85	1 day	no	0	N	0	Y	N	cerebral hemorrhage caused by car accident
S026	62	Male	22.50	Han Chinese	noodles	Shaanxi province	80	1 day	no	0	N	0	Y	N	cerebral hemorrhage after a high-altitude falling
S027	44	Male	22.60	Han Chinese	noodles,rice	Shaanxi province	85	1 day	no	0	N	0	N	N	cerebral infarction after an accidental fall
S028	40	Male	23.70	Han Chinese	noodles	Shaanxi province	80	1 day	no	0	N	0	Unknown	N	cerebral hemorrhage caused by car accident
S029	43	Male	24.10	Han Chinese	noodles	Shaanxi province	80	1 day	no	0	N	0	Unknown	N	cerebral hemorrhage caused by a fall from a residential building
S030	57	Male	23.90	Han Chinese	noodles	Shaanxi province	85	1 day	no	1	β-lactams	0	Unknown	N	cerebral hemorrhage caused by traumatic brain injury
S031	23	Female	21.20	Han Chinese	noodles,rice	Shaanxi province	90	1 day	no	0	N	0	Unknown	N	cerebral hemorrhage caused by car accident
S032	49	Male	24.20	Han Chinese	noodles	Shaanxi province	85	4 days	<1 week	1	β-lactams	0	Y	N	cerebral infarction after an accidental fall
S033	46	Male	23.00	Han Chinese	noodles	Shaanxi province	80	1 day	no	0	N	0	Y	N	cerebral hemorrhage caused by car accident

Table S2. Details of ASVs by Decontam removal method (>0.5% relative abundance in at least 1 negative control sample)

ASV_ID	Name	Negtive control		Real sample (M: mucosa; S: swap; L: lumen)						Ratio	
		mean(%)	norm$(%)	M, mean(%)	M, norm(%)	S, mean(%)	S, norm(%)	L, mean(%)	L, norm(%)	ratio: M/L	ratio: S/L
b46290a7e7e8a0	g__Propionibacterium	17.08	41.43	1.08E-01	3.29E+00	1.41E+00	1.19E+01	1.60E-02	1.27E+00	6.74	88.19
cad145f262b40d4	g__Phyllobacterium	6.12	24.75	1.81E-02	1.34E+00	1.01E-03	3.18E-01	4.12E-05	6.42E-02	438.77	24.58
892ac8f0ba4023e	g__Deinococcus	4.87	22.07	3.46E-04	1.86E-01	2.81E-02	1.68E+00	2.91E-04	1.71E-01	1.19	96.60
5dd8cfcb6737853	g__Corynebacterium	2.67	16.35	1.36E-02	1.17E+00	4.87E-03	6.98E-01	4.00E-04	2.00E-01	34.01	12.20
3c567b5989aacb(	g__Acinetobacter	2.31	15.19	3.55E-03	5.96E-01	4.02E-01	6.34E+00	5.65E-04	2.38E-01	6.29	710.69
d6ba4ffae4edfe7(	g__Enhydrobacter	2.21	14.88	3.53E-02	1.88E+00	1.57E+00	1.25E+01	1.15E-02	1.07E+00	3.08	137.38
b641ba20b982fc8	g__Finegoldia	1.43	11.95	4.55E-02	2.13E+00	1.23E-01	3.51E+00	1.35E-01	3.68E+00	0.34	0.91
159a95fa57a513(	g__Peptoniphilus	1.30	11.38	1.86E-03	4.31E-01	8.33E-03	9.13E-01	1.23E-02	1.11E+00	0.15	0.68
3b5747200a2264	g__Faecalibacterium;s__prausnitzii	1.17	10.83	1.67E-01	4.09E+00	NA	NA	NA	NA		
e270efd9d752f12	g__Deinococcus	1.15	10.74	1.95E-04	1.40E-01	4.18E-03	6.47E-01	5.63E-05	7.50E-02	3.47	74.35
015a52068e8c46	f__Enterobacteriaceae	0.99	9.95	NA	NA	NA	NA	NA	NA		
b864537803d53d	g__Chryseobacterium	0.89	9.42	1.12E-05	3.35E-02	NA	NA	0.00E+00	0.00E+00		
9e37ed6cd70126	f__Enterobacteriaceae	0.85	9.20	7.50E-01	8.66E+00	3.58E-01	5.98E+00	NA	NA		
508e4c73432b1e	g__Faecalibacterium;s__prausnitzii	0.78	8.81	3.25E-01	5.70E+00	NA	NA	NA	NA		
b9cadf71a31d7f6	g__Klebsiella	0.71	8.45	NA	NA	NA	NA	NA	NA		
4d2cd2e2b0522b	g__Ralstonia	0.67	8.21	3.97E-01	6.30E+00	3.70E-01	6.08E+00	NA	NA		
7ace17d08350e8	g__Faecalibacterium;s__prausnitzii	0.66	8.14	3.97E-01	6.30E+00	1.71E-01	4.14E+00	NA	NA		
ebab2ee0365303	g__Streptococcus	0.65	8.08	NA	NA	NA	NA	NA	NA		
cf46fd8225941f9	g__Porphyromonas	0.63	7.95	1.05E-02	1.03E+00	3.70E-01	6.08E+00	1.72E-02	1.31E+00	0.61	21.44
0e68bf8a5e7f297	f__Alcaligenaceae	0.63	7.95	1.20E-05	3.46E-02	1.94E-05	4.40E-02	NA	NA		
9a7cad3a10bfea2	g__Deinococcus	0.59	7.69	6.02E-04	2.45E-01	1.04E-01	3.23E+00	1.96E-03	4.43E-01	0.31	53.12
2891d7ed1ce769	f__Ruminococcaceae	0.57	7.56	NA	NA	NA	NA	NA	NA		
4e4b682bd22d3d	f__Enterobacteriaceae	0.56	7.49	4.52E-03	6.72E-01	6.22E-02	2.49E+00	1.97E-03	4.44E-01	2.29	31.57
d100ececcd20cd	g__Methanobrevibacter	0.55	7.42	3.49E-02	1.87E+00	2.80E-03	5.30E-01	5.31E-03	7.29E-01	6.58	0.53
a21b7c8a9c8c91	g__Paracoccus	0.51	7.14	1.01E-02	1.00E+00	3.69E-01	6.08E+00	9.92E-03	9.96E-01	1.02	37.22
b456e173f69fe31	g__Pseudomonas	0.51	7.14	5.89E-04	2.43E-01	2.87E-03	5.36E-01	2.87E-05	5.35E-02	20.54	100.14
52182c5c4a3c49	g__Bifidobacterium;s__adolescentis	0.51	7.14	2.66E-01	5.16E+00	5.19E-02	2.28E+00	NA	NA		
4875c832bb052f3	g__Corynebacterium	0.50	7.07	NA	NA	2.11E-03	4.59E-01	2.24E-05	4.74E-02		
64ecf598dba2bc2	g__Ruminococcus;s__bromii	0.49	7.00	NA	NA	5.84E-02	2.42E+00	NA	NA		
154b732b36acf6	f__Neisseriaceae	0.49	7.00	1.62E-05	4.03E-02	NA	NA	0.00E+00	0.00E+00		
d24fc605b43cdba	g__Staphylococcus	0.46	6.78	1.16E-02	1.07E+00	6.36E-01	7.98E+00	2.91E-03	5.39E-01	3.97	218.65
7c488e27a53405	f__Methylobacteriaceae	0.46	6.78	1.20E-02	1.10E+00	1.48E-02	1.21E+00	2.33E-03	4.83E-01	5.16	6.33
4805791b9b0b7b	g__Propionibacterium	0.43	6.55	0.00E+00	0.00E+00	2.64E-03	5.14E-01	NA	NA		
e250e857c8f9e5	g__Bifidobacterium	0.41	6.39	4.91E-01	7.01E+00	3.85E-01	6.21E+00	NA	NA		
f63810f9e4357d6	g__Ruminococcus	0.40	6.31	NA	NA	3.76E-03	6.14E-01	1.62E-02	1.27E+00		
15b36480ec074a	g__Micrococcus;s__luteus	0.37	6.06	4.22E-03	6.50E-01	2.18E-02	1.48E+00	7.32E-04	2.71E-01	5.77	29.85
86e9b54c2e0168	f__Micrococcaceae	0.37	6.06	NA	NA	1.66E-03	4.08E-01	4.80E-05	6.93E-02		
82adf095b346bca	g__Corynebacterium	0.37	6.06	4.10E-03	6.41E-01	6.69E-02	2.59E+00	3.55E-03	5.96E-01	1.16	18.85
36ef5b68fdf1ffb7	g__Fusobacterium	0.37	6.06	1.33E-02	1.15E+00	6.16E-01	7.85E+00	1.54E-02	1.24E+00	0.86	40.12
d426bdb86bf57d	g__Achromobacter	0.34	5.80	1.71E-02	1.31E+00	4.50E-03	6.71E-01	6.35E-03	7.97E-01	2.70	0.71
d61760ddf64834	g__Bacteroides;s__fragilis	0.33	5.71	3.85E-01	6.21E+00	3.06E-01	5.54E+00	NA	NA		
3841e7a970bb5a	g__Bifidobacterium;s__longum	0.32	5.62	6.74E-01	8.21E+00	2.28E-01	4.77E+00	NA	NA		
8b5f0e36d62361	g__Acinetobacter;s__schindleri	0.31	5.53	5.98E-03	7.74E-01	6.81E-04	2.61E-01	2.47E-04	1.57E-01	24.20	2.76
a081ff420ec7249	g__Ochrobactrum	0.29	5.34	4.59E-03	6.78E-01	1.59E-03	3.98E-01	3.78E-04	1.95E-01	12.14	4.19
bbb599007469c9	g__Mesorhizobium	0.29	5.34	1.51E-04	1.23E-01	8.54E-05	9.24E-02	NA	NA		
ea59ac5623e72b	g__Paracoccus	0.21	4.63	1.42E-03	3.77E-01	NA	NA	2.73E-04	1.65E-01	5.22	
6363e4f2bc2364	f__Xanthomonadaceae	0.20	4.52	2.80E-03	5.29E-01	1.44E-03	3.79E-01	1.36E-04	1.17E-01	20.53	10.55
51918b5cfae1a7	g__Microbispora;s__rosea	0.20	4.52	NA	NA	NA	NA	NA	NA		
4bae9869668a5b	g__Streptococcus	0.15	3.91	NA	NA	NA	NA	NA	NA		
efdf11aefe111b4	g__Acinetobacter;s__schindleri	0.14	3.78	1.27E-04	1.13E-01	NA	NA	NA	NA		
4b8fc883f410277	g__Anaerococcus	0.14	3.78	4.11E-04	2.03E-01	NA	NA	2.01E-02	1.42E+00	0.02	
5017c6fdb9693fb	g__Klebsiella	0.13	3.64	NA	NA	NA	NA	NA	NA		

$, normalized mean by arcsine transformation

Table S3. Effects (*F* score of PERMANOVA) of subject's characteristics on the microbiome communities

Organ	Region[$]	Age	BMI	Dietary (staple food)	Sampling duration after death (Min)	Antibiotic uses (Yes: 1; No: 0)	Hospitalization (Day)	High Blood Pressure	Cause of death	ICU (Yes: 1; No: 0)[#]
Skin	Left palm	0.99	1.39	2.2 (3.00e-03)	1.08	1.46	1.54 (1.40e-02)	1.27	0.96	3.02 (9.99e-04)
	Right palm	1.14	1.73 (1.80e-02)	0.97	0.76	1.27	1.15	0.88	1.19	1.12
	Back muscle	0.88	1.39	1.11	1.19	1	1.18	0.81	1.23	1.43
	Chest	1.72 (4.60e-02)	2.1 (8.99e-03)	0.64	1.24	0.59	1.17	0.99	1.1	1.11
	Left forearm	0.84	1.1	0.76	1.16	1.17	1.16	1.17	0.81	0.92
	Right forearm	0.63	0.88	0.71	0.92	1.58 (5.00e-02)	0.87	1.37	1.08	1.13
	Left calf	1.43	1.94 (1.40e-02)	1.28	0.76	1.58	1.34	0.94	0.98	1.51
	Right calf	1.01	0.92	1.43	0.67	1.13	1.23	1.41	0.85	1.47
	Left foot	1.2	1.18	1.04	1.46	0.92	1.06	1.11	0.92	1.01
	Right foot	0.65	1.1	0.74	1.74 (7.99e-03)	1.13	1.4 (3.50e-02)	1.22	1.2	1.31
Oral cavity	Left cheek	0.79	1.1	1.16	1.04	0.96	1.32 (2.30e-02)	1.48 (6.99e-03)	0.96	1.54 (2.00e-02)
	Right cheek	0.96	0.93	1.6 (2.50e-02)	0.8	1.08	1.38 (2.10e-02)	1.46 (9.99e-03)	1.04	1.79 (1.70e-02)
	Upper lip	1.18	0.81	1.47 (4.20e-02)	0.9	1.38	1.29	1.5 (1.80e-02)	1.21	1.19
	Lower lip	1.21	1.09	1.1	1.11	1.26	1.52 (1.10e-02)	1.15	1	2.08 (5.00e-03)
	Upper jaw	0.98	1.04	0.94	0.84	0.9	1.12	1.19	0.93	1.18
	Lower jaw	0.68	0.86	0.87	0.86	1.35	1.69 (2.00e-03)	1.13	1.47 (9.99e-04)	1.99 (3.00e-03)
Esophagus	Mid-esophagus, M	0.82	1.46 (3.40e-02)	1.35	0.99	1.21	1.22	1.05	1.01	1.13
	Up-dentate line, M	0.74	0.94	0.56	0.68	0.84	1.12	0.94	0.97	1.01
	Dentate line, M	1.01	1.13	0.85	1.26	0.79	0.71	0.88	1.08	0.61
	Down-dentate line, M	1.15	0.99	0.74	0.74	1.06	1.03	0.98	0.96	1.08
Stomach	Stomach fundus, M	0.98	0.9	0.65	0.48	1.06	1.49 (2.10e-02)	0.85	1.34 (2.80e-02)	1.46
	Stomach body, M	0.87	0.64	1.01	0.58	0.94	1.14	0.87	0.87	1.27
	Gastric juice, L	1.01	1.03	0.69	0.94	0.89	1.5 (1.60e-02)	1.14	1.11	1.43
	Stomach antrum, M	0.72	0.93	0.72	0.97	0.95	1.23	0.84	1.03	1.3
	Pylorus, M	0.79	1	0.74	1.11	1.01	1.25	1.05	1.09	1.39 (3.10e-02)
Small intestine	Superior-duodenum, M	1.42	1.47 (4.70e-02)	0.82	1	0.96	1.18	1.09	1.18	0.68
	Peri-duodenum, L	1.64 (4.60e-02)	1.32	1.01	1.07	1.07	1.44 (4.40e-02)	1.08	1.24	1.24
	Peri-duodenum, M	0.89	1.04	0.71	1	1.61	1.54	1.54 (4.50e-02)	0.63	1.54
	Flexor Ligament, M	1.05	0.9	1.08	0.89	1.19	1.2	0.88	1.08	1.13
	Jejunum 1m, L	1.09	1.46	1.61	1.22	0.78	1.63 (3.20e-02)	0.9	0.75	1.91 (4.40e-02)
	Jejunum 2m, M	0.89	0.91	0.92	0.81	0.81	1.02	1.1	1.17	0.88
	Ileum 3m, L	0.69	0.83	1.59	1.33	0.72	1.4	1.17	0.83	1.27
	Ileum 3m, M	0.83	1.35	0.99	0.99	1.05	1	1.32	1.07	1.07
	Ileocecal 1cm, L	0.9	1.44	1.27	0.54	1.44	1.03	1.52 (4.60e-02)	0.88	1.55
	Ileocecal 1cm, M	0.5	1.1	0.95	1.54	2.47 (7.99e-03)	0.96	1.12	1.33	1.09
	Ileocecal, L	1.42	1.03	1.08	1.14	1.11	1.41 (2.60e-02)	1.4 (4.00e-02)	1.12	1.9 (3.00e-03)
	Ileocecal, M	1.29	1.14	0.67	0.82	2.58 (2.00e-03)	1.44	0.82	1.02	2.63 (8.99e-03)
Appendix	Appendix, L	1.21	1.26	1.01	0.99	0.9	1.25	0.78	1.16	2.02 (1.50e-02)
Large intestine	Cecum, L	1.29	1.46 (3.80e-02)	0.83	1.21	0.8	1.42 (1.60e-02)	1.02	1.24 (2.90e-02)	1.99 (9.99e-04)
	Cecum, M	1.61	0.69	0.78	1.36	3.23 (5.00e-03)	2.41 (3.00e-03)	0.99	1.19	5.23 (9.99e-04)
	Ascending colon, L	0.97	1.09	1.13	1.06	1.39	1.1	0.83	1.07	1.18
	Ascending colon, M	1.18	1.04	0.78	0.86	3.6 (2.00e-03)	1.24	0.97	1.35 (3.80e-02)	2.71 (5.00e-03)
	Transverse colon, L	1.11	0.96	0.98	0.93	1.62 (1.80e-02)	1.44 (1.40e-02)	0.72	1.01	2.05 (9.99e-04)
	Transverse colon, M	0.85	1.31	1.25	0.76	2.93 (1.40e-02)	2.52 (5.00e-03)	0.8	1.25	3.98 (2.00e-03)
	Descending colon, L	1	1.23	1.18	1.21	1.14	1.77 (2.00e-03)	0.86	1.08	2.37 (9.99e-04)
	Descending colon, M	0.89	1.55	1.11	0.67	3.46 (4.00e-03)	2.32 (3.00e-03)	0.81	1.17	4.84 (9.99e-04)
	Signoid colon, L	1.01	0.89	1.15	0.8	0.88	1.71 (9.99e-04)	1	1.07	2.37 (2.00e-03)
	Signoid colon, M	2.41 (2.10e-02)	0.78	1.4	0.69	2.41 (1.40e-02)	1.54	1	1.22	2.21 (2.20e-02)
	Rectum, L	0.91	0.96	0.99	1.12	1.06	1.22	0.92	1.14	1.44 (4.40e-02)
	Rectum, M	0.71	0.83	1.36	0.71	2.48 (1.20e-02)	2.38 (2.00e-03)	0.8	1.24	4.74 (9.99e-04)
	Anus, L	0.84	0.96	1.33 (4.10e-02)	0.79	0.81	1.08	1.11	0.98	1.24

$, M: mucosa; L: lumen

#, The subjects who spent over 1 week of hospital stays all required ICU (**Table S1**), meaning that the effect of ICU was already present in the effect of length of hospital stays.

Table S4. PERMANOVA analysis of microbiome in each pair of organs.

Organ 1	Organ 2	*F*	R^2	*P*
Esophagus	Stomach	4.63	0.02	0.0009
Appendix	Small intestine	6.02	0.02	0.0009
Small intestine	Esophagus	11.46	0.02	0.0009
Large intestine	Appendix	12.18	0.03	0.0009
Appendix	Esophagus	12.22	0.08	0.0009
Appendix	Stomach	13.96	0.07	0.0009
Small intestine	Stomach	14.09	0.03	0.0009
Appendix	Skin	16.09	0.04	0.0009
Skin	Esophagus	20.11	0.04	0.0009
Skin	Stomach	25.43	0.05	0.0009
Appendix	Oral cavity	26.76	0.10	0.0009
Skin	Small intestine	35.09	0.05	0.0009
Stomach	Oral cavity	36.43	0.09	0.0009
Esophagus	Oral cavity	38.35	0.11	0.0009
Large intestine	Small intestine	50.62	0.06	0.0009
Large intestine	Esophagus	54.12	0.09	0.0009
Small intestine	Oral cavity	63.13	0.10	0.0009
Large intestine	Stomach	67.51	0.10	0.0009
Skin	Oral cavity	70.59	0.12	0.0009
Large intestine	Skin	121.93	0.14	0.0009
Large intestine	Oral cavity	139.51	0.18	0.0009

Table S5. Organ-specific microbial interactions shared between SECOM and SparCC method

N1	N2	Oral	Oral_isSpec	Esophagus	Esophagus_isSpec	Stomach	Stomach_isSpec	SI_M_JEJ	SI_M_JEJ_isSpec	SI_M_IIL	SI_M_IIL_isSpec	SI_L_JEJ	SI_L_JEJ_isSpec	SI_L_IIL	SI_L_IIL_isSpec	LI_M_RC	LI_M_RC_isSpec	LI_M_LC	LI_M_LC_isSpec	LI_L_RC	LI_L_RC_isSpec	LI_L_LC	LI_L_LC_isSpec	Method
k__Bacteria;p__Firmicutes;c__Clostridia;o__Clostridiales;f__Peptostreptococcaceae;g__Peptostreptococcus	k__Bacteria;p__Firmicutes;c__Bacilli;o__Gemellales;f__Gemellaceae;g__Gemella	-0.196	TRUE	0.52075	FALSE	0	FALSE	0	FALSE	0.3665	FALSE	0.309	FALSE	0.4713333	FALSE	0	FALSE	0	FALSE	0	FALSE	0	FALSE	sparcc
k__Bacteria;p__Firmicutes;c__Clostridia;o__Clostridiales;f__Peptostreptococcaceae;g__Peptostreptococcus	k__Bacteria;p__Firmicutes;c__Bacilli;o__Gemellales;f__Gemellaceae;g__Gemella	-0.248536	TRUE	0.3800673	TRUE	0	FALSE	0	FALSE	0	FALSE	0	FALSE	0	FALSE	0	FALSE	0	FALSE	0	FALSE	0	FALSE	secom
k__Bacteria;p__Firmicutes;c__Clostridia;o__Clostridiales;f__Peptostreptococcaceae;g__Peptostreptococcus	k__Bacteria;p__Firmicutes;c__Bacilli;o__Lactobacillales;f__Carnobacteriaceae;g__Granulicatella	-0.307	TRUE	0.57075	FALSE	0.423	FALSE	0	FALSE	0	FALSE	0.287	FALSE	0.3273333	FALSE	0	FALSE	0	FALSE	0	FALSE	0	FALSE	sparcc
k__Bacteria;p__Firmicutes;c__Clostridia;o__Clostridiales;f__Peptostreptococcaceae;g__Peptostreptococcus	k__Bacteria;p__Firmicutes;c__Bacilli;o__Lactobacillales;f__Carnobacteriaceae;g__Granulicatella	-0.344807	TRUE	0.5392314	TRUE	0	FALSE	0	FALSE	0	FALSE	0	FALSE	0	FALSE	0	FALSE	0	FALSE	0	FALSE	0	FALSE	secom
k__Bacteria;p__Proteobacteria;c__Gammaproteobacteria;o__Pasteurellales;f__Pasteurellaceae;g__Aggregatibacter	k__Bacteria;p__Firmicutes;c__Clostridia;o__Clostridiales;f__Peptostreptococcaceae;g__Peptostreptococcus	-0.415	TRUE	0.4823333	FALSE	0.644	FALSE	0	FALSE	0.296	FALSE	0.469	FALSE	0	FALSE	0	FALSE	0	FALSE	0	FALSE	0	FALSE	sparcc
k__Bacteria;p__Proteobacteria;c__Gammaproteobacteria;o__Pasteurellales;f__Pasteurellaceae;g__Aggregatibacter	k__Bacteria;p__Firmicutes;c__Clostridia;o__Clostridiales;f__Peptostreptococcaceae;g__Peptostreptococcus	-0.415147	TRUE	0.405995	FALSE	0.4767719	FALSE	0	FALSE	0	FALSE	0	FALSE	0	FALSE	0	FALSE	0	FALSE	0	FALSE	0	FALSE	secom
k__Bacteria;p__Firmicutes;c__Bacilli;o__Lactobacillales;f__Streptococcaceae;g__Streptococcus	k__Bacteria;p__Actinobacteria;c__Actinobacteria;o__Bifidobacteriales;f__Bifidobacteriaceae;g__Bifidobacterium	0	FALSE	-0.336	TRUE	0	FALSE	0	FALSE	0	FALSE	0.16	FALSE	0	FALSE	0.3733333	FALSE	0.274	FALSE	0.4196667	FALSE	0.3693333	FALSE	sparcc
k__Bacteria;p__Firmicutes;c__Bacilli;o__Lactobacillales;f__Streptococcaceae;g__Streptococcus	k__Bacteria;p__Actinobacteria;c__Actinobacteria;o__Bifidobacteriales;f__Bifidobacteriaceae;g__Bifidobacterium	-0.127892	FALSE	-0.451605	TRUE	-0.21422	FALSE	0	FALSE	0	FALSE	0	FALSE	0	FALSE	0.3683546	FALSE	0.2226852	FALSE	0.4109333	FALSE	0.5004317	FALSE	secom
k__Bacteria;p__Firmicutes;c__Clostridia;o__Clostridiales;f__Clostridiaceae;g__	k__Bacteria;p__Bacteroidetes;c__Bacteroidia;o__Bacteroidales;f__Bacteroidaceae;g__Bacteroides	0	FALSE	0.477	TRUE	0.26075	FALSE	0	FALSE	-0.215333	FALSE	0	FALSE	0	FALSE	-0.131	FALSE	-0.275	FALSE	-0.192	FALSE	-0.392667	FALSE	sparcc
k__Bacteria;p__Firmicutes;c__Clostridia;o__Clostridiales;f__Clostridiaceae;g__	k__Bacteria;p__Bacteroidetes;c__Bacteroidia;o__Bacteroidales;f__Bacteroidaceae;g__Bacteroides	0	FALSE	0.3715138	TRUE	0	FALSE	0	FALSE	-0.368303	FALSE	-0.196097	FALSE	0	FALSE	-0.108918	FALSE	-0.428932	FALSE	-0.171745	FALSE	-0.400955	FALSE	secom
k__Bacteria;p__Proteobacteria;c__Gammaproteobacteria;o__Enterobacteriales;f__Enterobacteriaceae;g__Klebsiella	k__Bacteria;p__Proteobacteria;c__Epsilonproteobacteria;o__Campylobacterales;f__Campylobacteraceae;g__Campylobacter	-0.3025	FALSE	0.4015	TRUE	0	FALSE	0	FALSE	0	FALSE	-0.2235	FALSE	-0.1375	FALSE	-0.203	FALSE	-0.212	FALSE	0	FALSE	-0.213333	FALSE	sparcc
k__Bacteria;p__Proteobacteria;c__Gammaproteobacteria;o__Enterobacteriales;f__Enterobacteriaceae;g__Klebsiella	k__Bacteria;p__Proteobacteria;c__Epsilonproteobacteria;o__Campylobacterales;f__Campylobacteraceae;g__Campylobacter	-0.423444	TRUE	0.3246988	TRUE	0	FALSE	0	FALSE	0	FALSE	0	FALSE	0	FALSE	0	FALSE	0	FALSE	0	FALSE	0	FALSE	secom
k__Bacteria;p__Firmicutes;c__Bacilli;o__Lactobacillales;f__Lactobacillaceae;g__Lactobacillus	k__Bacteria;p__Actinobacteria;c__Coriobacteriia;o__Coriobacteriales;f__Coriobacteriaceae;g__Eggerthella	0	FALSE	0	FALSE	0.1516667	FALSE	0	FALSE	0.4475	FALSE	-0.421	TRUE	0	FALSE	0	FALSE	-0.115	FALSE	0	FALSE	0.291	FALSE	sparcc
k__Bacteria;p__Firmicutes;c__Bacilli;o__Lactobacillales;f__Lactobacillaceae;g__Lactobacillus	k__Bacteria;p__Actinobacteria;c__Coriobacteriia;o__Coriobacteriales;f__Coriobacteriaceae;g__Eggerthella	0	FALSE	0	FALSE	0	FALSE	0	FALSE	0	FALSE	-0.407699	TRUE	0	FALSE	0	FALSE	0	FALSE	0.3300361	FALSE	0.2067015	FALSE	secom
k__Bacteria;p__Firmicutes;c__Clostridia;o__Clostridiales;f__Veillonellaceae;g__Megamonas	k__Bacteria;p__Firmicutes;c__Clostridia;o__Clostridiales;f__Clostridiaceae;g__	0	FALSE	0.263	FALSE	0	FALSE	0	FALSE	0.291	FALSE	0	FALSE	-0.465	TRUE	0.342	FALSE	0	FALSE	0.3155	FALSE	0	FALSE	sparcc
k__Bacteria;p__Firmicutes;c__Clostridia;o__Clostridiales;f__Veillonellaceae;g__Megamonas	k__Bacteria;p__Firmicutes;c__Clostridia;o__Clostridiales;f__Clostridiaceae;g__	0	FALSE	0	FALSE	0	FALSE	0	FALSE	0	FALSE	0	FALSE	0	FALSE	0.494859	FALSE	-0.293741	TRUE	0.2987459	FALSE	0	FALSE	secom
k__Bacteria;p__Firmicutes;c__Clostridia;o__Clostridiales;f__Lachnospiraceae;g__	k__Bacteria;p__Bacteroidetes;c__Bacteroidia;o__Bacteroidales;f__Bacteroidaceae;g__Bacteroides	0	FALSE	0.523	FALSE	0.49725	FALSE	0.626	FALSE	0.498	FALSE	0.7215	FALSE	0.523	FALSE	-0.165	TRUE	0	FALSE	0	FALSE	0.21	FALSE	sparcc
k__Bacteria;p__Firmicutes;c__Clostridia;o__Clostridiales;f__Lachnospiraceae;g__	k__Bacteria;p__Bacteroidetes;c__Bacteroidia;o__Bacteroidales;f__Bacteroidaceae;g__Bacteroides	0.3248587	FALSE	0.4692055	FALSE	0.4899766	FALSE	0.5516527	FALSE	0.6015372	FALSE	0.6031623	FALSE	0.5217677	FALSE	-0.261071	FALSE	-0.616336	TRUE	-0.368067	TRUE	-0.273083	FALSE	secom
k__Bacteria;p__Firmicutes;c__Clostridia;o__Clostridiales;f__Ruminococcaceae;g__Ruminococcus	k__Bacteria;p__Bacteroidetes;c__Bacteroidia;o__Bacteroidales;f__Porphyromonadaceae;g__Parabacteroides	0.4625	FALSE	0.449	FALSE	0.43575	FALSE	0.463	FALSE	0	FALSE	0.3215	FALSE	0	FALSE	-0.488	TRUE	-0.258	FALSE	-0.17	FALSE	0	FALSE	sparcc
k__Bacteria;p__Firmicutes;c__Clostridia;o__Clostridiales;f__Ruminococcaceae;g__Ruminococcus	k__Bacteria;p__Bacteroidetes;c__Bacteroidia;o__Bacteroidales;f__Porphyromonadaceae;g__Parabacteroides	0	FALSE	0.2975654	FALSE	0	FALSE	0	FALSE	0	FALSE	0.338307	FALSE	0	FALSE	-0.62722	TRUE	-0.271667	FALSE	-0.169352	FALSE	0	FALSE	secom
k__Bacteria;p__Firmicutes;c__Clostridia;o__Clostridiales;f__Lachnospiraceae;g__[Ruminococcus]	k__Bacteria;p__Actinobacteria;c__Actinobacteria;o__Bifidobacteriales;f__Bifidobacteriaceae;g__Bifidobacterium	0.364	FALSE	0.3365	FALSE	0.4165	FALSE	0.5584	FALSE	0	FALSE	0.298	FALSE	0	FALSE	-0.255	FALSE	-0.472	TRUE	0	FALSE	0	FALSE	sparcc
k__Bacteria;p__Firmicutes;c__Clostridia;o__Clostridiales;f__Lachnospiraceae;g__[Ruminococcus]	k__Bacteria;p__Actinobacteria;c__Actinobacteria;o__Bifidobacteriales;f__Bifidobacteriaceae;g__Bifidobacterium	0	FALSE	0.3143256	FALSE	0.2926352	FALSE	0.5080958	FALSE	0	FALSE	0.1949604	FALSE	0	FALSE	-0.181626	FALSE	-0.503942	TRUE	0	FALSE	0	FALSE	secom
k__Bacteria;p__Proteobacteria;c__Gammaproteobacteria;o__Enterobacteriales;f__Enterobacteriaceae;g__	k__Bacteria;p__Firmicutes;c__Clostridia;o__Clostridiales;f__Lachnospiraceae;g__	0.253	FALSE	0.314	FALSE	0.35	FALSE	0	FALSE	0.276	FALSE	0.289	FALSE	0.3386667	FALSE	0	FALSE	-0.453	TRUE	0	FALSE	0	FALSE	sparcc
k__Bacteria;p__Proteobacteria;c__Gammaproteobacteria;o__Enterobacteriales;f__Enterobacteriaceae;g__	k__Bacteria;p__Firmicutes;c__Clostridia;o__Clostridiales;f__Lachnospiraceae;g__	0.3288945	FALSE	0.2717289	FALSE	0.300091	FALSE	0.3817579	FALSE	0	FALSE	0.3145135	FALSE	0.3111304	FALSE	-0.249908	FALSE	-0.412462	TRUE	0	FALSE	-0.143624	FALSE	secom
k__Bacteria;p__Proteobacteria;c__Gammaproteobacteria;o__Enterobacteriales;f__Enterobacteriaceae;g__	k__Bacteria;p__Firmicutes;c__Clostridia;o__Clostridiales;f__Lachnospiraceae;g__Dorea	0.282	FALSE	0.37075	FALSE	0.2176667	FALSE	0.3846667	FALSE	0.3625	FALSE	0.201	FALSE	0.215	FALSE	0	FALSE	-0.361	TRUE	0.309	FALSE	0	FALSE	sparcc
k__Bacteria;p__Proteobacteria;c__Gammaproteobacteria;o__Enterobacteriales;f__Enterobacteriaceae;g__	k__Bacteria;p__Firmicutes;c__Clostridia;o__Clostridiales;f__Lachnospiraceae;g__Dorea	0	FALSE	0.3711064	FALSE	0	FALSE	0.2472364	FALSE	0	FALSE	0	FALSE	0.199633	FALSE	0	FALSE	-0.351906	TRUE	0	FALSE	0.3261647	FALSE	secom
k__Bacteria;p__Firmicutes;c__Clostridia;o__Clostridiales;f__Veillonellaceae;g__Veillonella	k__Bacteria;p__Firmicutes;c__Clostridia;o__Clostridiales;f__Lachnospiraceae;g__[Ruminococcus]	-0.2055	FALSE	0	FALSE	-0.176333	FALSE	0	FALSE	-0.221	FALSE	0	FALSE	0	FALSE	-0.12	FALSE	0.7	TRUE	-0.306	FALSE	0.219	FALSE	sparcc
k__Bacteria;p__Firmicutes;c__Clostridia;o__Clostridiales;f__Veillonellaceae;g__Veillonella	k__Bacteria;p__Firmicutes;c__Clostridia;o__Clostridiales;f__Lachnospiraceae;g__[Ruminococcus]	0	FALSE	-0.405472	FALSE	-0.313124	FALSE	0	FALSE	0	FALSE	0	FALSE	0	FALSE	0	FALSE	0.5975555	TRUE	0	FALSE	0.2493052	FALSE	secom
k__Bacteria;p__Proteobacteria;c__Gammaproteobacteria;o__Enterobacteriales;f__Enterobacteriaceae;g__	k__Bacteria;p__Firmicutes;c__Clostridia;o__Clostridiales;f__Ruminococcaceae;g__Faecalibacterium	0.2253333	FALSE	0.433	FALSE	0.2073333	FALSE	0.4816667	FALSE	0.251	FALSE	0.2785	FALSE	0.2896667	FALSE	0	FALSE	-0.453	TRUE	0	FALSE	0	FALSE	sparcc
k__Bacteria;p__Proteobacteria;c__Gammaproteobacteria;o__Enterobacteriales;f__Enterobacteriaceae;g__	k__Bacteria;p__Firmicutes;c__Clostridia;o__Clostridiales;f__Ruminococcaceae;g__Faecalibacterium	0	FALSE	0	FALSE	0	FALSE	0	FALSE	0	FALSE	0	FALSE	0.2824688	TRUE	-0.40471	FALSE	0	FALSE	-0.28601	FALSE	0	FALSE	secom
k__Bacteria;p__Bacteroidetes;c__Bacteroidia;o__Bacteroidales;f__Rikenellaceae;g__	k__Bacteria;p__Actinobacteria;c__Actinobacteria;o__Bifidobacteriales;f__Bifidobacteriaceae;g__Bifidobacterium	0.155	FALSE	0.384	FALSE	0.4113333	FALSE	0	FALSE	0.232	FALSE	0	FALSE	0	FALSE	0	FALSE	0.239	FALSE	-0.348	TRUE	0	FALSE	sparcc
k__Bacteria;p__Bacteroidetes;c__Bacteroidia;o__Bacteroidales;f__Rikenellaceae;g__	k__Bacteria;p__Actinobacteria;c__Actinobacteria;o__Bifidobacteriales;f__Bifidobacteriaceae;g__Bifidobacterium	0	FALSE	0	FALSE	0	FALSE	0	FALSE	0	FALSE	0	FALSE	0	FALSE	0	FALSE	0.1807661	TRUE	-0.422769	TRUE	0	FALSE	secom
k__Bacteria;p__Bacteroidetes;c__Bacteroidia;o__Bacteroidales;f__Bacteroidaceae;g__Bacteroides	k__Bacteria;p__Actinobacteria;c__Coriobacteriia;o__Coriobacteriales;f__Coriobacteriaceae;g__Collinsella	0	FALSE	0.4585	FALSE	0.3513333	FALSE	0.288	FALSE	0.759	FALSE	0.347	FALSE	0	FALSE	-0.148	FALSE	0	FALSE	-0.265	TRUE	0	FALSE	sparcc
k__Bacteria;p__Bacteroidetes;c__Bacteroidia;o__Bacteroidales;f__Bacteroidaceae;g__Bacteroides	k__Bacteria;p__Actinobacteria;c__Coriobacteriia;o__Coriobacteriales;f__Coriobacteriaceae;g__Collinsella	0	FALSE	0.5026011	TRUE	0	FALSE	0	FALSE	0	FALSE	0.2955591	FALSE	0	FALSE	0	FALSE	0	FALSE	-0.420474	FALSE	-0.33989	FALSE	secom
k__Bacteria;p__Proteobacteria;c__Betaproteobacteria;o__Burkholderiales;f__Alcaligenaceae;g__Sutterella	k__Bacteria;p__Actinobacteria;c__Coriobacteriia;o__Coriobacteriales;f__Coriobacteriaceae;g__Eggerthella	0	FALSE	0.3225	FALSE	0.2675	FALSE	0.3703333	FALSE	0	FALSE	0.2525	FALSE	0.2345	FALSE	0	FALSE	0	FALSE	-0.3515	TRUE	0	FALSE	sparcc
k__Bacteria;p__Proteobacteria;c__Betaproteobacteria;o__Burkholderiales;f__Alcaligenaceae;g__Sutterella	k__Bacteria;p__Actinobacteria;c__Coriobacteriia;o__Coriobacteriales;f__Coriobacteriaceae;g__Eggerthella	0	FALSE	0	FALSE	0	FALSE	0	FALSE	0	FALSE	0.3360984	TRUE	0	FALSE	0	FALSE	0	FALSE	-0.403759	FALSE	-0.37226	FALSE	secom
k__Bacteria;p__Firmicutes;c__Clostridia;o__Clostridiales;f__Ruminococcaceae;g__Ruminococcus	k__Bacteria;p__Bacteroidetes;c__Bacteroidia;o__Bacteroidales;f__Bacteroidaceae;g__Bacteroides	0.3255	FALSE	0.53875	FALSE	0.54325	FALSE	0.582	FALSE	0	FALSE	0.564	FALSE	0	FALSE	0	FALSE	0	FALSE	-0.22	TRUE	0	FALSE	sparcc
k__Bacteria;p__Firmicutes;c__Clostridia;o__Clostridiales;f__Ruminococcaceae;g__Ruminococcus	k__Bacteria;p__Bacteroidetes;c__Bacteroidia;o__Bacteroidales;f__Bacteroidaceae;g__Bacteroides	0	FALSE	0.4381691	FALSE	0.5146902	FALSE	0.4425076	FALSE	0	FALSE	0.4612082	FALSE	0	FALSE	0	FALSE	-0.46305	TRUE	-0.267577	FALSE	-0.27881	FALSE	secom
k__Bacteria;p__Firmicutes;c__Bacilli;o__Lactobacillales;f__Streptococcaceae;g__Streptococcus	k__Bacteria;p__Bacteroidetes;c__Bacteroidia;o__Bacteroidales;f__Prevotellaceae;g__Prevotella	0	FALSE	0.5436667	FALSE	0.44225	FALSE	0.4284	FALSE	0	FALSE	0.611	FALSE	0	FALSE	0	FALSE	0	FALSE	-0.2975	TRUE	0	FALSE	sparcc
k__Bacteria;p__Firmicutes;c__Bacilli;o__Lactobacillales;f__Streptococcaceae;g__Streptococcus	k__Bacteria;p__Bacteroidetes;c__Bacteroidia;o__Bacteroidales;f__Prevotellaceae;g__Prevotella	-0.191743	FALSE	0.4977678	FALSE	0.4622725	FALSE	0.4175167	FALSE	-0.171408	FALSE	0.5674747	FALSE	0.2661423	FALSE	0.2561913	FALSE	-0.338035	TRUE	0	FALSE	0	FALSE	secom
k__Bacteria;p__Firmicutes;c__Clostridia;o__Clostridiales;f__Veillonellaceae;g__Veillonella	k__Bacteria;p__Bacteroidetes;c__Bacteroidia;o__Bacteroidales;f__Prevotellaceae;g__Prevotella	0.3636667	FALSE	0.48475	FALSE	0.70125	FALSE	0.6478	FALSE	0.262	FALSE	0.545	FALSE	0.424	FALSE	0.2475	FALSE	0	FALSE	-0.315	TRUE	-0.239333	TRUE	sparcc
k__Bacteria;p__Firmicutes;c__Clostridia;o__Clostridiales;f__Veillonellaceae;g__Veillonella	k__Bacteria;p__Bacteroidetes;c__Bacteroidia;o__Bacteroidales;f__Prevotellaceae;g__Prevotella	0.2937627	FALSE	0.3433698	FALSE	0.7128606	FALSE	0	FALSE	0	FALSE	0.4732668	FALSE	0	FALSE	0	FALSE	0	FALSE	0	FALSE	-0.189033	TRUE	secom
k__Bacteria;p__Fusobacteria;c__Fusobacteriia;o__Fusobacteriales;f__Fusobacteriaceae;g__Fusobacterium	k__Bacteria;p__Bacteroidetes;c__Bacteroidia;o__Bacteroidales;f__Prevotellaceae;g__Prevotella	0.3536667	FALSE	0.523	FALSE	0.4785	FALSE	0.53275	FALSE	0.307	FALSE	0.452	FALSE	0	FALSE	0	FALSE	0.1995	FALSE	-0.312	TRUE	0.275	FALSE	sparcc
k__Bacteria;p__Fusobacteria;c__Fusobacteriia;o__Fusobacteriales;f__Fusobacteriaceae;g__Fusobacterium	k__Bacteria;p__Bacteroidetes;c__Bacteroidia;o__Bacteroidales;f__Prevotellaceae;g__Prevotella	0.2633381	FALSE	0.579366	FALSE	0.4620082	FALSE	0	FALSE	0	FALSE	0.4007155	FALSE	0	FALSE	-0.50342	TRUE	0	FALSE	-0.346349	FALSE	0.1422495	FALSE	secom
k__Bacteria;p__Fusobacteria;c__Fusobacteriia;o__Fusobacteriales;f__Fusobacteriaceae;g__Fusobacterium	k__Bacteria;p__Firmicutes;c__Clostridia;o__Clostridiales;f__Clostridiaceae;g__	-0.124	FALSE	0	FALSE	0	FALSE	0	FALSE	0	FALSE	-0.212	FALSE	0	FALSE	0.3543333	FALSE	0	FALSE	0.5735	TRUE	-0.27	FALSE	sparcc
k__Bacteria;p__Fusobacteria;c__Fusobacteriia;o__Fusobacteriales;f__Fusobacteriaceae;g__Fusobacterium	k__Bacteria;p__Firmicutes;c__Clostridia;o__Clostridiales;f__Clostridiaceae;g__	0	FALSE	-0.296435	FALSE	-0.521324	FALSE	0	FALSE	0	FALSE	-0.399276	FALSE	0.2315969	FALSE	0.3071068	FALSE	-0.320635	FALSE	0.5223644	TRUE	-0.167266	FALSE	secom
k__Bacteria;p__Firmicutes;c__Clostridia;o__Clostridiales;f__;g__	k__Bacteria;p__Bacteroidetes;c__Bacteroidia;o__Bacteroidales;f__Bacteroidaceae;g__Bacteroides	0.2993333	FALSE	0.5473333	FALSE	0.53325	FALSE	0.605	FALSE	0.4085	FALSE	0.681	FALSE	0.45925	FALSE	0	FALSE	-0.171	FALSE	-0.102	FALSE	-0.296	TRUE	sparcc
k__Bacteria;p__Firmicutes;c__Clostridia;o__Clostridiales;f__;g__	k__Bacteria;p__Bacteroidetes;c__Bacteroidia;o__Bacteroidales;f__Bacteroidaceae;g__Bacteroides	0.4029626	FALSE	0	FALSE	0	FALSE	0	FALSE	0	FALSE	0.6181771	TRUE	0.3874167	FALSE	0	FALSE	-0.313522	FALSE	-0.217529	FALSE	-0.271666	FALSE	secom
k__Bacteria;p__Firmicutes;c__Clostridia;o__Clostridiales;f__Lachnospiraceae;g__Dorea	k__Bacteria;p__Bacteroidetes;c__Bacteroidia;o__Bacteroidales;f__Rikenellaceae;g__	0.253	FALSE	0.40625	FALSE	0	FALSE	0.4465	FALSE	0.2903333	FALSE	0.4285	FALSE	0.415	FALSE	0	FALSE	0	FALSE	0	FALSE	-0.290333	TRUE	sparcc
k__Bacteria;p__Firmicutes;c__Clostridia;o__Clostridiales;f__Lachnospiraceae;g__Dorea	k__Bacteria;p__Bacteroidetes;c__Bacteroidia;o__Bacteroidales;f__Rikenellaceae;g__	0	FALSE	0.4507722	TRUE	0	FALSE	0	FALSE	0.2582162	FALSE	0	FALSE	0	FALSE	-0.481416	FALSE	-0.405203	FALSE	-0.296538	FALSE	-0.467124	FALSE	secom
k__Bacteria;p__Firmicutes;c__Erysipelotrichi;o__Erysipelotrichales;f__Erysipelotrichaceae;g__[Eubacterium]	k__Bacteria;p__Bacteroidetes;c__Bacteroidia;o__Bacteroidales;f__Rikenellaceae;g__	0	FALSE	0.407	FALSE	0	FALSE	0.3586	FALSE	0.372	FALSE	0.2595	FALSE	0	FALSE	0	FALSE	0.253	FALSE	0.334	FALSE	-0.3305	TRUE	sparcc
k__Bacteria;p__Firmicutes;c__Erysipelotrichi;o__Erysipelotrichales;f__Erysipelotrichaceae;g__[Eubacterium]	k__Bacteria;p__Bacteroidetes;c__Bacteroidia;o__Bacteroidales;f__Rikenellaceae;g__	0	FALSE	0.3894213	FALSE	0	FALSE	0	FALSE	0	FALSE	0	FALSE	0	FALSE	0	FALSE	0	FALSE	0.2258968	FALSE	-0.341751	TRUE	secom
k__Bacteria;p__Firmicutes;c__Clostridia;o__Clostridiales;f__Lachnospiraceae;g__[Ruminococcus]	k__Bacteria;p__Firmicutes;c__Clostridia;o__Clostridiales;f__;g__	0	FALSE	0.408	FALSE	0.401	FALSE	0	FALSE	0.2853333	FALSE	0.545	FALSE	0.40375	FALSE	0	FALSE	0.3745	FALSE	0	FALSE	-0.4235	TRUE	sparcc
k__Bacteria;p__Firmicutes;c__Clostridia;o__Clostridiales;f__Lachnospiraceae;g__[Ruminococcus]	k__Bacteria;p__Firmicutes;c__Clostridia;o__Clostridiales;f__;g__	0	FALSE	0.3863307	FALSE	0.3451279	FALSE	0	FALSE	0.2601367	FALSE	0.4418571	FALSE	0.4050251	FALSE	-0.47302	TRUE	0	FALSE	0	FALSE	-0.485532	TRUE	secom
k__Bacteria;p__Firmicutes;c__Clostridia;o__Clostridiales;f__Ruminococcaceae;g__	k__Bacteria;p__Firmicutes;c__Clostridia;o__Clostridiales;f__Lachnospiraceae;g__Dorea	0.347	FALSE	0.54575	FALSE	0.3205	FALSE	0.6128	FALSE	0.6393333	FALSE	0.685	FALSE	0.3865	FALSE	0.242	FALSE	0	FALSE	0.2766667	FALSE	-0.205	TRUE	sparcc
k__Bacteria;p__Firmicutes;c__Clostridia;o__Clostridiales;f__Ruminococcaceae;g__	k__Bacteria;p__Firmicutes;c__Clostridia;o__Clostridiales;f__Lachnospiraceae;g__Dorea	0	FALSE	0.5076653	FALSE	0.310285	FALSE	0.519312	FALSE	0.5505452	FALSE	0.6321582	FALSE	0.4365915	FALSE	-0.226748	FALSE	-0.302493	FALSE	0	FALSE	-0.408442	TRUE	secom

Supplementary Methods

Guidelines for organ donation after cardiac death in China

I. Purposes

To regulate organ donation after cardiac death (DCD) and protect the legitimate rights of organ donors, the China Organ Donation Committee (CODC) has formulated the “Regulations on Human Organ Transplantation”. The purpose of a national unified DCD guideline is to establish an ethical and operational procedure and put forward legal, ethical, as well as medically acceptable suggestions for the implementation of DCD on the basis of respecting the rights of patients, to avoid any possible harm to patients, their families, recipients, and medical care team.

II. Scope of use

This guide applies to clinical staff involved in organ donation procedures that specifically occur in hospitals. However, during the specific implementation of controlled DCD, adjustments can be made based on the characteristics of patients, families, or regions. These guidelines should not replace a physician's clinical judgement.

III. Definition and classifications of DCD

Definition: Organ donation performed by subjects after cardiac death. In the past, it was also called non-heart beating donation (NHBD).

Classifications: Currently, the DCD classification defined by the Maastricht (Maastricht) International Conference in the Netherlands in 1995 is generally used worldwide. Classification V has recently been proposed as a supplement to the other four classifications.

Maastricht classification:

Category I: Dead in the out-of-hospital setting. Those who died before admission, warm ischemia time is unknown. Uncontrolled.

Category II: Unsuccessful resuscitation. Those who fail cardiopulmonary resuscitation. These patients are usually given timely cardiopulmonary resuscitation when the heart stops beating, and the warm ischemia time is known. Uncontrolled.

Category III: Awaiting cardiac arrest. Dying patients awaiting cardiac arrest after planned

withdrawal of supportive care-warm ischemia time is known. Controlled

Category IV: Cardiac arrest while brain death. Cardiac arrest in confirmed brain-dead donors. Sometimes patients have agreed to donate and are waiting for organ procurement personnel to arrive. Warm ischemia time is known and may be limited. Controlled.

Category V: Unexpected cardiac arrest in critically ill patients. Warm ischemia time is known and may be limited. Uncontrolled.

IV. Personnel and responsibilities of participating in DCD

Personnel involved in DCD include attending *physicians*, organ donation coordinators, organ procurement organizations (OPO) members and related auxiliary personnel, hospital donation committee personnel, etc. The personnel of DCD group participate in the DCD process and get the procedure working fluently. Formal meetings should be held at key points to clarify responsibilities.

1. *Attending physician*: participate in the entire donation except for organ removal. Mainly responsible for screening potential donors, making preliminary assessments to determine donors, contacting the provincial organ donation committee (PODC), and submitting basic patient information. Communicate with families to withdraw cardiopulmonary support, the specific implementation process, and announcing death. Assist the organ donation coordinator to discuss organ donation issues with families; provide medical intervention to patients; fill in DCD records, organize case reviews, and report to the hospital donation committee for filing.
2. *Organ Donation Coordinator*: Mainly responsible for discussing organ donation issues with families and obtaining legal documents such as informed consent for donation. Organ donation coordinators are trained and qualified by the Red Cross Organization.
3. *OPO team*: Mainly responsible for organ harvesting and not involved in the removal of supportive care.
4. *Personnel of the hospital donation committee or ethics committee:* supervise whether the donation process complies with the principles of informed consent,

whether the legal documents are complete, the reporting of DCD documents, and record management.

5. *Other personnel*: including anesthesiologists required for organ harvesting, mainly assist the OPO team in completing the organ harvesting.

V. DCD procedures and key points

1. Screening potential donors

1.1 Requirements for potential organ donors: (a) Severe neurological injury and/or other organ failure requiring mechanical ventilation or circulatory support, refering to the United States Network for Organ Resource Sharing (UNOS) evaluation standards for preliminary evaluation. (b) Families request to withdraw supportive treatment (refer to spouse, adult sons/daughters, parents, or legally authorized personnel).

After initial screening a potential organ donor by the attending physician, he or she should conduct a consultation and discussion to make it clear that the patient's prognosis is poor and death is unavoidable.

1.2 Decision to withdraw supportive care: The attending physician informs the family of the patient's condition and inevitable death. The family decides to withdraw supportive care after fully understanding and accepting the patient's condition. Discussion regarding cardiopulmonary support withdrawal and organs/tissues donation should be separated, which can alleviate the family's grief and avoid the conflicts of interest related to care of the patient.

1.3 Contact PODC: contact PODC, consult the OPO team, submit the patient's basic documents, and evaluate whether the patient conforms to the minimum standards for donation.

2. Preliminary assessment

2.1 Assessing the feasibility of DCD: Once the decision is made to withdraw cardiopulmonary support, the attending physician needs to consider the possibility of DCD. If the patient meets the donation criteria and is expected to die within 60 minutes after cardiopulmonary support is withdrawn, DCD can be considered, and a preliminary evaluation should be conducted by the attending physician before obtaining informed consent from the family for organ and tissue donation. If certain tests must be performed during the prediction process, the attending physician

should inform the families and record in detail the conversation and the families' informed consent. DCD cannot be performed if the attending physician predicts that the patient can survive for more than 60 minutes after cardiopulmonary support withdrawal.

2.2 *Formal submission to PODC*: All potential organ donors should be notified to the PODC. PODC assigns an organ donation coordinator to identify donation wishes. The organ donation coordinator should conduct in-depth interviews with the patient's families and clearly explain the specific process and requirements of DCD to the families. When the attending physician obtains oral informed consent regarding DCD from the patient's family, the organ donation coordinator should discuss all issues related to DCD with the patient's family before obtaining written informed consent. PODC should assign an OPO team to be responsible for organ harvesting.

3. Informed consent

3.1 Discuss donation-related issues with families: Organ donation should be part of high-quality end-of-life medical care, and the question of donation should be raised with all patients who may be candidates for donation. Medical staffs of various professions should discuss the issues of DCD and withdrawal of treatment with the families. After agreement of organ donation, the families should be informed in detail the relevant issues. If the families propose organ donation on their own before making the decision to withdraw cardiopulmonary support, or if the patient proposes a donation intention before waking up, it must be recorded in detail in the medical record and further discussed with the families based on the patient's clinical condition.

3.2 *Acquirement of Informed consent*. After fully understanding organ donation, the families should sign a formal informed consent with the organ donation coordinator. Doctors should record in detail discussions with families. If a family member objects to a known potential donor's willingness to donate, the opinion should be respected.

3.3 Report and record: Submit DCD documents to the hospital donation committee or ethics committee for filing. The hospital donation committee or ethics committee will be responsible for supervising the organ donation process, assessing whether legal procedures such as informed consent are complete, and reporting to the provincial organ donation office (PODO).

4. Donor management and comprehensive assessment

Extensive interventions including comprehensive assessment and medical intervention should be conducted to prepare the tissue and organs for the patients. This intervention is in favor of the benefits of potential recipients, and it should be complied with informed consent and the principle of harmlessness. medical intervention can only be carried out with the informed consent of the awaked patient or immediate families. Measures that can alleviate patient suffering should not be restricted or reduced, and measures that hasten patient death should not be applied. Premortem intervention should be performed by the attending physician. A comprehensive assessment should include the patient's basic information, detailed personal history, past medical history, and laboratory tests. Effective medical intervention should be applied as far as possible with clear evidence. If there is insufficient evidence to prove its effectiveness, if there is no illegal operation and the informed consent of the family is obtained, it can be implemented under the careful choice of the attending physician. All interventions applied must be documented in detail.

5. Withdrawal of cardiopulmonary support and declaration of death

5.*1 Withdrawal of cardiopulmonary support:* The organ harvesting or transplant team cannot be involved in the withdrawal of supportive care. If the patient's families wish to be present when cardiopulmonary support is withdrawn, their request should be respected. Once death of the patient is declared, the families should leave immediately. The attending physician and organ donation coordinator are responsible for providing comfort and taking care of the families. The death process should be videotaped, and drugs that accelerate the death should not be used. The time of withdrawal of cardiorespiratory support should be accurately recorded. The patient's vital signs, including heart rate, respiratory rate, blood pressure, blood oxygen saturation, and urine output, should be recorded every minute after cardiopulmonary support is withdrawn. Accurately record warm ischemia time, which refers to the period from removal of the tracheal intubation to tracheal cold perfusion.

5.*2. Declaration of Death:* Criteria for cardiac death: arrest of circulation, loss of reflexes, loss of heartbeat and pulse, loss of respiration. Due to the time limit of DCD, detection or inspection needs to be used to determine the cessation of circulation quickly and accurately. If possible, invasive arterial blood pressure testing and ultrasound can be used for confirmation. An ECG is not required to determine death because electrical activity may be present for several minutes after circulatory

arrest. To determine whether the circulatory arrest is irreversible or permanent, it should be observed for a few minutes before the declaration of death. The observation time is at least 2 minutes, but not more than 5 minutes. Death should be announced by 2 or more attending physicians, the time of death should be accurately recorded, and the death process shall be videotaped (the transplant surgeons or OPO team should not be present). Organ harvesting can be performed after the declaration of death, and once death is declared, measures cannot be taken to restore circulation. To prevent inhalation and secondary lung injury, re-intubation is allowed.
If there is no irreversible circulatory arrest at a specific time after the removal of cardiopulmonary support, the patient should return to the pre-arranged area for continued end-of-life care.

6. Organ removal

Once the death is declared, the OPO team can intervene and start the resection as soon as possible to shorten the warm ischemia time as far as possible. Before resection, the surgical team and Personnel inside the Operating Room should be coordinated prepare for resection. The operation start time, intubation and perfusion time, each donated organ resection time and operation end time should be accurately recorded.

7. Case review and summary

A case review should be conducted after each completion of DCD, and relevant documents should be compiled and reported to the hospital donation committee (or ethics committee) and PODC for record management.

Approval system for living organ transplantation

To safely, effectively and reasonably protect the life, health, interests and dignity of living organ donors and organ transplant patients, we must comply with key regulations including "Regulations on Human Organ Transplantation", "Provisions of the Ministry of Health on Regulating Living Organ Transplantation", "Human Organ Transplantation" as well as "Management of Clinical Application of Organ Transplantation Technology" based on the approval system for living organ transplantation.

I. Review principles

Members of the Organ Transplantation Ethics Committee should abide by the principles of minimal harm to the donor, self-help, informed consent, free of charge, equality, and fairness, conduct organ transplantation ethics review work based on the principle of safeguarding the rights and interests of both donors and recipients, and avoid formalities. The focus of the review includes: a) the authenticity of the hospital where the organ donor donated the organ; b) whether there is any sale or disguised sale of human organs c) whether the matching of organs and the adaptions of the recipient comply with ethical principles and technical management standards for human organ transplantation.

II. Formal review

Documents related to living organ transplantation declared by the application department will be formally reviewed by the secretary of the Ethics Committee for the Clinical Application of Human Organ Transplantation Technology (referred to Ethics Committee) in accordance with the "Provisions of the Ministry of Health on Regulating Living Organ Transplantation". In case the unqualified documents, the applicant department is required to supplement or correct the documents in a timely manner. For those who fail the formal review due to serious mistakes of application department (incompatible donor and recipient blood types, etc.), the related department and individual will be held responsible and further be punished by the ethics committee or the hospital.

III. Verification of documents: For application materials that conform to the regulations and requirements after formal review, the secretary of the ethics committee will review and approve the relevant contents of the application materials one by one:

1. With the assistance of the application department, interview the intended recipients of living organ transplants, living organ donors and their relatives, review and approve relevant materials and information.

2. Adhere to the principle of back-to-back, and declare to the donor, recipient, and families one by one whether the situation reflected in the materials is consistent with their own wishes. Consult

with the immediate families about the informed consent of donor’s donation, and preserve the audio and video data.

3. After interviewing the recipients and donors of living organ transplants and their relatives, the secretary of the ethics committee will verify and record the results from the relevant departments based on the household registration, certificate of relationship and other materials of the application materials.

4. Use the second-generation ID card verification system to check the original ID card in the application materials to identify its authenticity.

IV. Convene an ethics committee meeting

1. After verifying the application materials, the secretary of the ethics committee should report the verification situation to the chairman of the ethics committee or the deputy chairman entrusted by the chairman in a timely manner, and ask for instructions whether to hold an ethics meeting on living organ transplantation.

2. Upon decision to convene an ethics meeting on living organ transplantation, the secretary of the ethics committee should inform all ethics committee members in advance of the time and place of the meeting.

3. Ethics committee meetings are hosted by the chairman, and mainly focus on reviewing and discussing the following matters:

3.1. Whether the materials provided by the living organ donor and the recipient are true and legal, and whether their relationship complies with the "Provisions of the Ministry of Health on Regulating Living Organ Transplantation". Only spouses (limited to spouses who have been married for more than three years or have children after marriage), lineal relatives, kinship relationships formed due to assistance (limited to the relationship between adoptive parents and children, and the relationship between stepparents and children).

3.2 Whether it is the real intention of organ donor

3.3 Whether there is any types of transactions for organs

3.4 Whether the donor matching and the recipient’s according to the technical regulations for human organ transplantation.

3.5 Whether the physical and psychological conditions of living organ donors and beneficiaries

meet medical standards for organ donation and transplantation.

3.6 Scientifically and comprehensively assess the possible impact of organ donors, and assure no significant physical or mental damage to donors through the donation and the surgical removal of the organs.

3.7 Whether the donation complies with medical and ethical principles.

3.8 Whether there are suitable donation surgeries and treatments, as well as medical plans for pre-transplantation preparation and post-transplantation treatment.

V. Committee Resolutions and Operation implementation

1. After reviewing and discussing the application for living organ transplantation, all committee members have to agree on and provide a written approval for organ donation and transplantation, which will be signed by the chairman and sent to the implementing department. The ethics committee should reply to the department and relevant parties in writing with the main reasons for declination of organ donation and transplantation.

2. After the ethics committee issues a written approval for carrying out organ donation and transplant surgery, the hospital should submit supporting documents to the Shaanxi Provincial Health and Family Planning Commission for further review, and the comments should be forwarded to relevant departments in a timely manner by the hospital ethics committee.

3. After organ transplantation operation, the medical staff responsible should submit a report on the operation implementation to the ethics committee within 48 hours.

4. The ethics committee should archive ethics committee meeting record, organ transplantation application documents, written comments on agreeing or disagreeing with organ transplantation, postoperative reports and other relevant materials.

VI. Others

(1) All permission of organ transplant cases approved by the ethics committee validate for one year after approval. If the operation has not been performed within one year, the department must prepare updated data and submit an explanation to the ethics committee for review and approval by the ethics committee.

(2) If a organ transplant approved by the ethics committee is terminated due to alteration of the

donor's intention, physical condition of recipient which should be reported to the ethics committee for recordation.

(3) The implementing department must submit a summary of organ transplantation this year to the Organ Transplantation Ethics Committee at the end of the each year, which includes: statistics on the number of live surgeries, prognosis of recipients, reasons for recipients failed to undergo surgery, etc.
