# Supplementary material for: Defining the biogeographical map and potential bacterial translocation of microbiome in human ‘surface organs’
Source: Nat Commun. 2024 Jan 10;15:427. doi: 10.1038/s41467-024-44720-6 (PMC10781665; doi:10.1038/s41467-024-44720-6)
Supplement: Supplementary file 3 — Description of Additional Supplementary Files [file 41467_2024_44720_MOESM3_ESM.pdf]

# Description of Additional Supplementary Files

**Supplementary Data 1**: Pathway abundance in each organ.

**Supplementary Data 2**: Organ-specific microbial interactions based on SECOM method.
