# Supplementary material for: Defining the biogeographical map and potential bacterial translocation of microbiome in human ‘surface organs’
Source: Nat Commun. 2024 Jan 10;15:427. doi: 10.1038/s41467-024-44720-6 (PMC10781665; doi:10.1038/s41467-024-44720-6)
Supplement: Supplementary file 6 — Reporting Summary [file 41467_2024_44720_MOESM6_ESM.pdf]

nature portfolio

Corresponding author(s): Jun Yu, Jun-jun She

Last updated by author(s): Jun 6, 2023

Reporting Summary

Nature Portfolio wishes to improve the reproducibility of the work that we publish. This form provides structure for consistency and transparency in reporting. For further information on Nature Portfolio policies, see our Editorial Policies and the Editorial Policy Checklist.

Statistics

For all statistical analyses, confirm that the following items are present in the figure legend, table legend, main text, or Methods section.

n/a	Confirmed	
☐	☒	The exact sample size (*n*) for each experimental group/condition, given as a discrete number and unit of measurement
☐	☒	A statement on whether measurements were taken from distinct samples or whether the same sample was measured repeatedly
☐	☒	The statistical test(s) used AND whether they are one- or two-sided<br>*Only common tests should be described solely by name; describe more complex techniques in the Methods section.*
☒	☐	A description of all covariates tested
☐	☒	A description of any assumptions or corrections, such as tests of normality and adjustment for multiple comparisons
☐	☒	A full description of the statistical parameters including central tendency (e.g. means) or other basic estimates (e.g. regression coefficient) AND variation (e.g. standard deviation) or associated estimates of uncertainty (e.g. confidence intervals)
☐	☒	For null hypothesis testing, the test statistic (e.g. *F*, *t*, *r*) with confidence intervals, effect sizes, degrees of freedom and *P* value noted<br>*Give P values as exact values whenever suitable.*
☒	☐	For Bayesian analysis, information on the choice of priors and Markov chain Monte Carlo settings
☒	☐	For hierarchical and complex designs, identification of the appropriate level for tests and full reporting of outcomes
☐	☒	Estimates of effect sizes (e.g. Cohen's *d*, Pearson's *r*), indicating how they were calculated

Our web collection on statistics for biologists contains articles on many of the points above.

Software and code

Policy information about availability of computer code

Data collection	Illumina NovaSeq platform<br>PacBio RS II system
Data analysis	R software (version 3.6.3); SMRT Link (v6.0); lima (v1.7.1); cutadapt (v1.9); QIIME2 (version 2019.4.0); decontam (v1.6.0); aod (v1.3.1); ALDEx2 (1.80.0); SparCC (Jun 20, 2011); Cytoscape (version 3.7.1); PICRUSt2 (v2.4.0); ANCOM-BC2 (v2.2.2); SECOM (v2.2.2)

For manuscripts utilizing custom algorithms or software that are central to the research but not yet described in published literature, software must be made available to editors and reviewers. We strongly encourage code deposition in a community repository (e.g. GitHub). See the Nature Portfolio guidelines for submitting code & software for further information.

Data

Policy information about availability of data

All manuscripts must include a data availability statement. This statement should provide the following information, where applicable:

- Accession codes, unique identifiers, or web links for publicly available datasets
- A description of any restrictions on data availability
- For clinical datasets or third party data, please ensure that the statement adheres to our policy

All the raw sequencing data generated in this study have been deposited in NCBI Sequence Read Archive (SRA) under BioProject PRJNA1049979. ASV sequences

were classified taxonomically using Greengenes database at 99% identity cut-off. Source data are provided with this paper. Remaining data are available within the Article or Supplementary Information.

Research involving human participants, their data, or biological material

Policy information about studies with human participants or human data. See also policy information about sex, gender (identity/presentation), and sexual orientation and race, ethnicity and racism.

Reporting on sex and gender	Written informed consent was obtained from each enrolled donor via next-of-kin consent to permit the collection and banking of samples. The study was approved by the Clinical Application Ethics Committee of First Affiliated Hospital of Xi'an Jiaotong University (Approval No. XJTU1AF2019LSK-059) and conducted in accordance with the Declaration of Helsinki. The age and sex have been documented in Table S1. Sex of participants (n=33) was determined based on assigned. As we only have two female donors, we are not able to evaluate the effect of donor sex on the composition of microbiome. The higher number of males recruited compared to females was because of the much lower incidence of unnatural deaths among females. At our hospital, we receive more potential male donors that suffer accidental deaths, e.g., vehicle accidents, risk-taking activities and occupational risks (e.g., construction workers) (Table S1)
Reporting on race, ethnicity, or other socially relevant groupings	To minimize variation in microbial community caused by diet, all human donors were Chinese and were recruited from the northwest China, where the diet is heavily dominated by carbohydrates with middle portion of protein (Table S1).
Population characteristics	This study comprised of 31 males and 2 females with age 49.5 ± 10.5 (years; mean ± SD) and BMI 23.3 ± 1.5 (mean ± SD)
Recruitment	All the human donors were declared dead by cardiovascular death. Subjects were excluded if they had tumour, infectious disease, or metabolic disease.
Ethics oversight	The study was approved by the Clinical Application Ethics Committee of First Affiliated Hospital of Xi'an Jiaotong University (Approval No. XJTU1AF2019LSK-059) and conducted in accordance with the Declaration of Helsinki.

Note that full information on the approval of the study protocol must also be provided in the manuscript.

Field-specific reporting

Please select the one below that is the best fit for your research. If you are not sure, read the appropriate sections before making your selection.

☒ Life sciences ☐ Behavioural & social sciences ☐ Ecological, evolutionary & environmental sciences

For a reference copy of the document with all sections, see nature.com/documents/nr-reporting-summary-flat.pdf

Life sciences study design

All studies must disclose on these points even when the disclosure is negative.

Sample size	Sample size (1608 samples from 33 individuals) were decided based on the accepted standards in the filed of study and the experience from similar study in our laboratory (Liu et al. Gastroenterology. 2021;160(7):2395-2408.)
Data exclusions	Subjects were excluded if they had tumour, infectious disease, or metabolic disease.
Replication	Collection of multi-site from 7 surface organs of oral cavity, esophagus, stomach, small intestine, appendix, large intestine and skin for all 33 human subjects were successfully conducted. For ASVs co-existed in all upper GI or lower GI organs intra-individually, this phenomena can be replicated in >50% of subjects (prevalence >50%).
Randomization	All samples were analyzed randomly.
Blinding	The authors who performed the microbiome analysis were initially made blinded to the organ collection followed by unblinding after completion of microbiome data extraction, upon which correlation and data extrapolation were made based on the subject's metadata

Reporting for specific materials, systems and methods

We require information from authors about some types of materials, experimental systems and methods used in many studies. Here, indicate whether each material, system or method listed is relevant to your study. If you are not sure if a list item applies to your research, read the appropriate section before selecting a response.

Materials & experimental systems

n/a	Involved in the study
☒	☐ Antibodies
☒	☐ Eukaryotic cell lines
☒	☐ Palaeontology and archaeology
☒	☐ Animals and other organisms
☒	☐ Clinical data
☒	☐ Dual use research of concern
☒	☐ Plants

Methods

n/a	Involved in the study
☒	☐ ChIP-seq
☒	☐ Flow cytometry
☒	☐ MRI-based neuroimaging
